# Optimization of an efficient solid-phase enrichment medium for *Salmonella* detection using response surface methodology

**DOI:** 10.1186/s13568-019-0819-0

**Published:** 2019-06-28

**Authors:** Feng Tang, Zhi Chen, Feng Wang, Hongyan Hou, Weiyong Liu, Han Xiao, Jiao Hu, Yan Xiong, Hui Zhang, Zhongju Chen, Hanming Peng, Jun Lu, Wanjun Luo, Ying Zhao, Miao Lin

**Affiliations:** 1 Department of Laboratory Medicine, Wuhan Children’s Hospital (Wuhan Maternal and Child Healthcare Hospital), Tongji Medical College, Huazhong University of Science & Technology, Wuhan, 430016 People’s Republic of China; 2 Microbiological Laboratory, Wuhan Center for Disease Control and Prevention, Wuhan, 430015 People’s Republic of China; 3 Department of Laboratory Medicine, Tongji Hospital, Tongji Medical College, Huazhong University of Science and Technology, Wuhan, 430030 People’s Republic of China; 4 Biobank, Wuhan Children’s Hospital (Wuhan Maternal and Child Healthcare Hospital), Tongji Medical College, Huazhong University of Science & Technology, Wuhan, 430016 People’s Republic of China; 5 Institute of Environmental Safety, Wuhan Academy of Agricultural Sciences, Wuhan, 430072 People’s Republic of China; 6 Microbiological Laboratory, Qiaokou District Center for Disease Control and Prevention, Wuhan, 430030 People’s Republic of China; 7 Gastroenterology Department, Wuhan Children’s Hospital (Wuhan Maternal and Child Healthcare Hospital), Tongji Medical College, Huazhong University of Science & Technology, Wuhan, 430016 People’s Republic of China; 8 Microbiological Laboratory, Jianghan District Center for Disease Control and Prevention, Wuhan, 430030 People’s Republic of China; 9 Hospital Acquired Infection Control Department, Wuhan Children’s Hospital (Wuhan Maternal and Child Healthcare Hospital), Tongji Medical College, Huazhong University of Science & Technology, Wuhan, 430016 People’s Republic of China; 10 Key Laboratory of Analytical Chemistry for Biology and Medicine (Ministry of Education), College of Chemistry and Molecular Sciences, State Key Laboratory of Virology, Wuhan University, Wuhan, 430072 People’s Republic of China

**Keywords:** *Salmonella*, Visual detection, Solid-phase enrichment assay, Hydrogen sulfide, Response surface methodology

## Abstract

*Salmonella* is a pathogenic bacterium contributing to food poisoning and acute infectious intestinal disease. The traditional standard detection method is based on the principle of liquid phase enrichment and has a low sensitivity on targeted bacteria. We previously developed a visual immunosensor technique for efficient detection and isolation of *Salmonella* by applying fluorescent nanobioprobes on a specially-designed cellulose-based swab. In this study, a whole-sample solid-phase enrichment assay (WSEA) was established by optimization of the enrichment medium using response surface methodology (RSM), a powerful statistical tool for regression analysis. The optimal formula was determined as: 0.60% polyvalent poly peptone, 0.40% buffered peptone water, 0.09% ferric citrate amine, 0.24% sodium hyposulfite, 0.035% cystine, 0.01 µg mL^−1^ super absorbing polymer, 0.011% sodium deoxycholate, 15.00 µg mL^−1^ ethyl green and 30.00 µg mL^−1^ sodium selenite. Using this formula, *Salmonella* was visualized with naked eyes by relying on the indication of black spots formed on the swab. The analytic sensitivity of the assay was determined as 10^1^ cells mL^−1^ with a concentration of interfering bacteria (*Escherichia coli*) at 10^5^ cells mL^−1^. This optimized formula was confirmed with 4006 patients’ fecal samples, in which the positive rate was 0.42% by the conventional culture-based method and 2.12% by WSEA. The optimized formulation on solid phase enrichment by RSM allows relatively quick, low-cost, and large-scale detection of *Salmonella*, and could be used in grassroots medical institutions.

**Electronic supplementary material:**

The online version of this article (10.1186/s13568-019-0819-0) contains supplementary material, which is available to authorized users.

## Introduction

*Salmonella* is a pathogenic bacterium contributing to food poisoning and acute infectious intestinal disease (LaRock et al. 2015; Scallan et al. 2011). *Salmonella enterica* can be divided into two main groups, typhoidal and non-typhoidal *Salmonella*. The global health impact of non-typhoidal *Salmonella* is high, with an estimated 93.8 million illnesses and 155,000 deaths each year (Majowicz et al. 2010). The typhoidal *Salmonella* causes more than 22 million infections and 216,000 deaths per year in developing countries (Jin et al. 2017). In China, *Salmonella* was the most common pathogen causing food poisoning according to the annual bulletins of National Health and Family Planning Commission of China. In the US, *Salmonella* was also responsible for foodborne illnesses, and approximately 42,000 cases of serious salmonellosis were reported annually. However, only 7554 cases were confirmed by culture diagnostic test and 618 cases by culture-independent diagnostic test in 2016 (Marder et al. 2017).

The traditional standard detection method is based on liquid phase enrichment, which has not achieved sufficient sensitivity on bacterial enrichment and high isolation rate on *Salmonella* (Bell et al. 2016). A large number of false negative cases (*Salmonella* carriers) become potential sources of infection. Though some rapid detection technologies on culture-independent diagnostic test are emerging, the greatest challenge is still to obtain pure isolates, which is a prerequisite for the etiological diagnosis, molecular epidemiological analysis, and antibiotic sensitivity experiments (Langley et al. 2015; Marder et al. 2017).

In our previous study, a novel immunosensor technique for efficient detection and isolation of *Salmonella* was established by applying fluorescent nanobioprobes on a specially-designed cellulose-based swab (Tang et al. 2016). However, this technique is not suitable for large-scale detection due to two major reasons. One is that it requires expensive instrument, such as laser scanning confocal microscope, which may not be available in many hospitals. Another reason is that it is complex to synthesize and prepare the specialized bio-probe reagent, especially at large-scales. In this study, a whole-sample solid-phase enrichment assay (WSEA) was further established through appropriate optimization and verification of the enrichment medium.

As a sulfate-reducing bacterium, *Salmonella* can reduce cystine or sodium thiosulfate and produce hydrogen sulfide (H_2_S) (Jackson et al. 2013), which reacts with ammonium ferric citrate to produce ferrous sulfide (Gong et al. 2014). At relatively high concentrations, ferrous sulfide can form visible black spots on the surface of the specially designed cellulose-based swab. Accordingly, these essential components (cystine, sodium hyposulfite and ferric citrate amine) are the most important variables of the chromogenic reaction. In contrast to *Salmonella*, the H_2_S reaction of *Escherichia coli* is negative. A more important factor that affects the sensitivity and specificity of this chromogenic reaction is the incubation medium, which is required not only to enhance the growth of *Salmonella* and inhibit the growth of non-*Salmonella*, but also to help the conversion of hydrogen sulfide to ferrous sulfide. It is difficult to evaluate and optimize these multiple factors of the medium.

Optimization of parameters by the conventional method involves changing one independent variable while unchanging all others at a fixed level. This is extremely time-consuming and expensive for a large number of variables and also may result in wrong conclusions (Tam et al. 2012). Response surface methodology (RSM) is a mathematical model that is often used to predict the response of a system to multifactor condition and evaluate the relationship among a set of controllable experimental factors and observed results. Therefore, the RSM could be used to predict the optimized medium from multiple factors (Li et al. 2018; Verma et al. 2018). RSM has been also applied in biotechnology, biopharmaceuticals, food industry, medical applications, and other fields (Abd Elrazak et al. 2013; Zhang et al. 2012). The central composite design (CCD) in RSM is an empirical statistical technique for modeling complex systems, evaluating the simultaneous effects of several factors, and searching optimum conditions for desirable responses (Mousavi et al. 2018; Dastkhoon et al. 2017). In this study, CCD was applied to optimize the formulation of the chromogenic medium for a wider applicability on different *Salmonella* serotypes. Multiple variables were applied to the mathematical model to predict the optimal formula, which was then evaluated using standard strains and confirmed using human fecal samples. We concluded that our optimized WSEA is an efficient and low-cost method for large-scale detection for *Salmonella*. It may have broad applications, especially in the grassroots medical institutions, with its low cost and ease of use.

## Materials and methods

To optimize the solid-phase enrichment medium for *Salmonella* detection, we firstly used the CCD in RSM to mathematically predict the possible optimized media. Nine standard *Salmonella* strains belonging to eight major types of A-F (O:2–O:11) group were applied to one model of statistical regression analysis. In order to enhance the practicality of the method, 40 *Salmonella* strains belonging to 40 serotypes (O:2–O:11) from local patients were also collected and applied to another model. *E. coli* ATCC 25922 was used as a non-*Salmonella* control. These strains are listed in Table 1. The CCD system automatically generated 20 combinations from variables for enhanced production of ferrous sulfide. According to these combinations, the results of visual observation were recorded accordingly, and then the predicted response was automatically calculated. Analysis of variance (ANOVA) in CCD was used to evaluate the statistical significance and the goodness-of-fit of the model. Subsequently, the mixtures of *Salmonella* and *E. coli* strains were used to test the analytic sensitivity of the new assay. Ultimately, the optimized formula was confirmed with 4006 human fecal samples.

**Table 1:** Bacterial strains used in this study

Bacterial strains	Source
Standard strains	
*Salmonella paratyphoid* A CMCC 50093	CMCC
*Salmonella enteritidis* ATCC 13076	ATCC
*Salmonella typhi* ATCC 19430	ATCC
*Salmonella senftenberg* CMCC 50105	CMCC
*Salmonella typhimurium* ATCC 14028	ATCC
*Salmonella infanti* ATCC 51741	ATCC
*Salmonella manhattan* CMCC 50152	CMCC
*Salmonella aberdeen* CMCC 50147	CMCC
*Salmonella muenster* CCAM 090010	CCAM
Local strains	
*Salmonella typhi* *Salmonella agona* *Salmonella derby* *Salmonella anatum* *Salmonella enteritidis* *Salmonella sinstorf* *Salmonella butantan* *Salmonella aberdeen* *Salmonella braenderup* *Salmonella london* *Salmonella risen* *Salmonella isangi* *Salmonella muenster* *Salmonella potsdam* *Salmonella kentucky* *Salmonella meleagridis* *Salmonella cremieu* *Salmonella saintpaul* *Salmonella kottbus* *Salmonella typhimurium* *Salmonella manhattan* *Salmonella papuana* *Salmonella singapore* *Salmonella mikawasima* *Salmonella newport* *Salmonella thompson* *Salmonella duboin* *Salmonella brijbhumi* *Salmonella gallinarum*-*pullorum* *Salmonella pakistan* *Salmonella blockley* *Salmonella senftenberg* *Salmonella schwarzengrund* *Salmonella mbandaka* *Salmonella stanley* *Salmonella eppendorf* *Salmonella sandiego* *Salmonella newlands* *Salmonella paratyhi A* *Salmonella paratyhi B*	All local strains were from the Wuhan Centers for Disease Control and Prevention
Non-*Salmonella* strain	
*Escherichia coli* (*E. coli*) ATCC 25922	ATCC

*ATCC* American Type Culture Collection, *CMCC* National Center for Medical Culture Collections, *CCAM* Collection Center of Agricultural Microbiology

### Ethics

The protocol of this study was approved by the Human Research Advisory Committees of the Wuhan Center for Disease Control and Prevention (WHCDCIRB-K-2018016) and the Wuhan Children’s Hospital of Tongji Medical College of Huazhong University of Science & Technology for Medical Sciences ([2018]IEC(S140)). All procedures performed in studies involving human participants were in accordance with the ethical standards of the relevant national and institutional committees on human experimentation and with the Helsinki Declaration of 1975, revised in 2008. All participants were voluntary and agreed to participate in the methodological comparative study. The medical reports were still based on the results of the national standard test method.

### Production of swabs

**Fig. 1:**
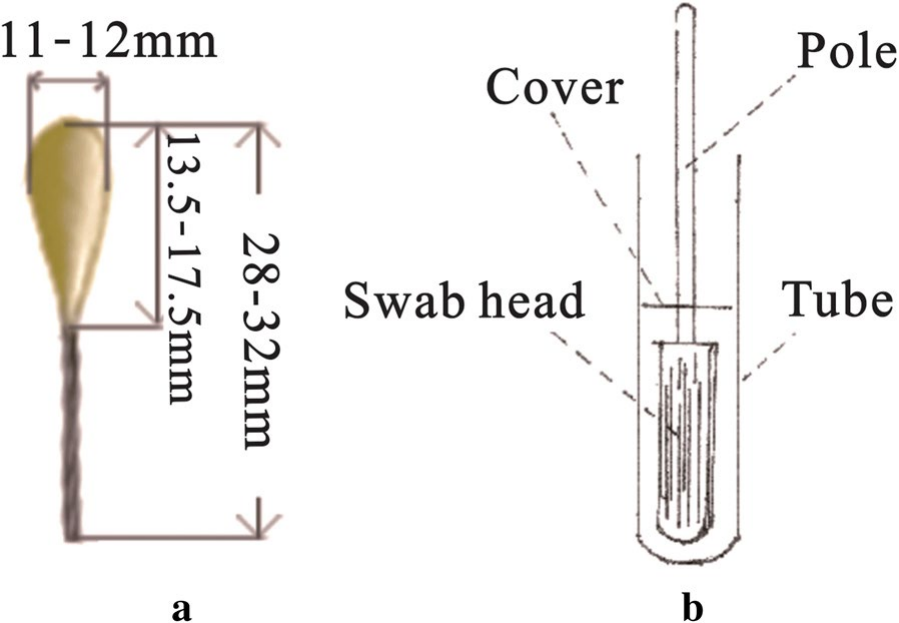
A schematic diagram of the swab: **a** the size of the swab, and **b** the swab immersed in the sample tube, which is covered with a silicone plug to prevent contamination

Each swab is 28.0–32.0 mm long and 11.0–12.0 mm wide with a 14.5 cm bamboo handle (Fig. 1a). It contains 0.18 g of 100% medical-grade degreasing cotton, which has the ability to absorb approximately 1000 μL of medium or sample solution. The swabs were sterilized by ethylene oxide in a sterilizer (900 mg/L, 55–60 °C, 6 h), and not treated with any reagents (e.g. formaldehyde or decolorizer). The swabs were immersed with sufficient medium at 25 °C, then dried under sterile conditions at room temperature. When applied to culture, the sample tube was covered with a silicone plug (Fig. 1b).

### Detection procedures

The bacterial samples were proportionally prepared with 10^1^ cells mL^−1^ of *Salmonella* and 10^5^ cells mL^−1^ of *E. coli*. The swabs were immersed with 1000 μL of test samples at 25 °C, and then cultured for 16–24 h at 37 °C. From the 18th h to the 24th h of culture, these swabs were examined with naked eyes once every 3 h. The swabs without black spots at 24 h were excluded as non-*Salmonella* samples. The black spots on swabs were picked up with a small sterile iron hook (15 cm length handle and 2 mm elbow) and then plated onto the xylose lysine desoxycholate (XLD) agar and modified semi-solid rappaport vassiliadis (MSRV) agar plates for isolation of positive colonies, which were incubated for 18–24 h at 37 °C. In this study, XLD agar, nutrient broth and MSRV agar were purchased from Qingdao Hi-Tech Industrial Park Hope Bio-Technology Co., Ltd. China.

### Response surface methodology (RSM) analysis of the medium used on swab

Nine standard *Salmonella* strains and 40 *Salmonella* strains from local patients belong to 40 serotypes and were divided into two groups as simultaneous models. The three key variables for enhanced production of ferrous sulfide included 5% cystine, 20% sodium hyposulfite, and 10% ferric citrate amine. The basic nutrients and inhibiting ingredients used in the study contain ethyl green 15.00 μg, sodium selenite 30.00 μg, 60.00 μL of 10% the polyvalent polypeptone, 40.00 μL of 10% buffered peptone water, and 11.00 μL of 1% sodium deoxycholate, and 0.01 μg of super absorbing polymer in 600.00 μL of distilled water. In addition, 20% sodium carbonate was used to adjust the pH to 7.2, and then made up 1.00 mL total with distilled water. In this study, ferric citrate amine, sodium hyposulfite and cystine were purchased from Shanghai Chemical Reagent Co., Ltd. China. Super absorbing polymer (Product number: 127284) is a polymer material based on polyacrylamide with good water absorption and water-holding capacity, and was purchased from Liaocheng City Yongxing Environmental Protection Material Co., Ltd. China.

The CCD in Design-Expert statistical software (RSM, Version 8.0.6.1; Stat-Ease Inc, Minneapolis, MN, USA) automatically generates 20 combinations from the three key factors. Data were fitted into a quadratic polynomial model to obtain optimal regression coefficients (Additional file 1). The relationships between factors and responses were evaluated by fitting the following second-order polynomial model as previously described (Li et al. 2018):

1 $$Y_{i} = \beta_{0} + \sum {\beta_{\iota } x_{i} + \sum {\beta_{ij} } } x_{i} x_{j} + \sum {\beta_{jj} x_{j}^{2} }$$

where Y_i_ is the predicted response, β_0_ is the coefficient for intercept, β_i_ is the coefficient of linear effect, β_ij_ is the coefficient of interaction effect, β_jj_ is the coefficient of quadratic effect, and χ_i_ and χ_j_ are the coded independent variables. The three variables we used for the optimization include 10% ferric citrate amine (X_1_), 20% sodium hyposulfite (X_2_), and 5% cystine (X_3_). The predicted responses for nine standard *Salmonella* strains (Y_1_) and for 40 *Salmonella* strains from local patients (Y_2_) were derived from Eq. (1) (Li et al. 2018). ANOVA was used to evaluate the statistical significance and the goodness-of-fit of the model. The significance of every variable in the model was evaluated by the corresponding P-value. P < 0.05 was considered statistically significant.

### Analytic sensitivity of the assay

Analytic sensitivity, which is also known as the limit of detection, refers to the minimum amount of analyte that can be detected on a certain background levels (Clark et al. 2009). After the optimization formula is obtained, mixtures of standard *Salmonella* strains (at a concentration of 10^1^ cells mL^−1^) and *E. coli* (at a concentration of 10^4^ cells mL^−1^, 10^5^ cells mL^−1^ or 10^6^ cells mL^−1^) were used to test the analytic sensitivity of our assay. They were diluted with sterile water to obtain serial dilutions, which were counted in duplicates on agar plates. Serial dilutions of bacteria were prepared using a turbidity meter (DensiCHEK Plus, BioMerieux). The prepared swabs were inserted into the above test tubes and cultured at 37 °C. The number of swabs that produced black spots were recorded in the corresponding time period (18–19 h, 23–24 h, 35–36 h, 47–48 h). Black spots were picked *in situ* as soon as possible. The subsequent identification procedures were in accordance with the International Commission of Microbiological Specializations on Food (ICMSF) (ICMSF 2011).

### Evaluation of the optimized medium on human feces samples

Human fecal samples were collected using a disposable sampling stick, which was gently inserted into the volunteer’s anus and repeatedly rotated to obtain suitable feces. Then the sampling stick was submerged in 2.00 mL of purified water and mixed well. A total of 4006 samples were collected and detected for *Salmonella* at the Wuhan Center for Disease Control and Prevention of China. Each sample was divided into two tubes (1000 μL per tube); one tube was detected by the conventional culture-based method according to standard ICMSF identification (ICMSF 2011), the other by the solid phase enrichment method. The swab was directly applied to sampling, culture, and isolation following the detection procedures as described earlier. The suspected black spots were isolated *in situ* onto XLD agar plates and identified using standard ICMSF identification (Andrews 1994; ICMSF 2011).

## Results

### Optimization of the medium used in WSEA

Based on the confirmation report from CCD operation and the above second-order polynomial model (Eq. 1), the Design-Expert of statistical software was employed to perform the stepwise regressions of experimental results from CCD (Table 2) to generate two quadratic polynomial models below:

2 $${\text{Y1}} = 9 7. 3 5+ 10. 7 7 {\text{X}}_{ 1} + 9. 2 2 {\text{X}}_{ 2} + 6. 3 1 {\text{X}}_{ 3} - 0. 8 4 {\text{X}}_{ 1} {\text{X}}_{ 2} + 1.0 1 {\text{X}}_{ 1} {\text{X}}_{ 3} - 2. 70{\text{X}}_{ 2} {\text{X}}_{ 3} - 1 1. 6 7 {\text{X}}_{1}^{2} - 7.0 9 {\text{X}}_{2}^{2} - 5. 7 8 {\text{X}}_{3}^{2}$$

3 $${\text{Y2}} = 9 8. 1 8+ 1 1.0 3 {\text{X}}_{ 1} + 8. 8 9 {\text{X}}_{ 2} + 6. 3 7 {\text{X}}_{ 3} - 2. 50{\text{X}}_{ 1} {\text{X}}_{ 2} + 0. 6 3 {\text{X}}_{ 1} {\text{X}}_{ 3} - 3. 7 5 {\text{X}}_{ 2} {\text{X}}_{ 3} - 1 1. 6 6 {\text{X}}_{1}^{2} - 8. 5 7 {\text{X}}_{2}^{2} - 5. 4 8 {\text{X}}_{3}^{2}$$

**Table 2:** Predicted responses to central composite design with three independent variables

Run	10% ferric citrate amine (μL)	20% sodium hyposulfite (μL)	5% cystine (μL)	Response 1 (%)	Response 2 (%)
1	9.00	12.00	7.00	96.30	97.50
2	6.00	16.00	10.00	74.07	75.00
3	14.05	12.00	7.00	85.19	85.00
4	9.00	12.00	7.00	100.00	97.50
5	6.00	8.00	4.00	52.50	47.50
6	9.00	12.00	7.00	92.59	97.50
7	6.00	8.00	10.00	66.67	65.00
8	12.00	8.00	4.00	70.37	70.00
9	3.95	12.00	7.00	37.04	40.00
10	9.00	12.00	12.05	92.59	92.50
11	12.00	16.00	4.00	85.19	85.00
12	12.00	16.00	10.00	92.60	90.00
13	6.00	16.00	4.00	74.07	72.50
14	9.00	18.73	7.00	96.29	92.50
15	12.00	8.00	8.00	85.19	90.00
16	9.00	12.00	1.95	62.96	67.50
17	9.00	12.00	7.00	96.30	100.00
18	9.00	12.00	7.00	100.00	100.00
19	9.00	12.00	7.00	100.00	97.50
20	9.00	5.27	7.00	51.85	50.00

Response 1 means the isolation rate of the 9 standard *Salmonella*; Response 2 means the isolation rate of 40 local *Salmonella* strains

The variables were coded according to the above equations. The CCD system automatically generated 20 combinations from the three variables and the predicted responses are listed in Table 2. The values were rounded for experimental convenience. Then the enrichment effects of 20 kinds of media on target bacteria were analyzed according to the production rate of hydrogen sulfide. ANOVA analysis showed that both Y1 (Additional file 2) and Y2 (Additional file 3) had a high adequate precision (Adeq P) value (Y1 = 10.77, Y2 = 15.40) (Table 3).

**Table 3:** ANOVA analysis of the predicted responses for quadratic polynomial models

Group	Equation	Source	SS	DF	MS	F-value	Prob > F
Y1	CV% = 8.69	Model (Y1)	6080.75	9	675.64	13.76	0.0002
	R^2^ = 0.9253	Residual	491.00	10	49.10		
	AdjR^2^ = 0.8580	Lack-of-fit	445.27	5	89.05	9.74	0.0130
	PredR^2^ = − 0.4747	Pure error	45.73	5	9.15		
	AdeqP = 10.773	Total	6571.75	19			
Y2	CV% = 6.56	Model (Y2)	6418.45	9	713.16	25.47	< 0.001
	R^2^ = 0.9582	Residual	279.98	10	28.00		
	AdjR^2^ = 0.9206	Lack-of-fit	271.65	5	54.33	32.60	0.0008
	PredR^2^ = 0.6907	Pure error	8.33	5	1.67		
	AdeqP = 15.404	Total	6698.44	19			

*SS* sum of squares, *DF* degrees of freedom, *MS* mean squared, *CV%* variation coefficient, *R*^*2*^ determination coefficient, *AdjR*^*2*^ adjusted R-squared, *PredR*^*2*^ predR-squared, *A deqP* adequate precision

**Fig. 2:**
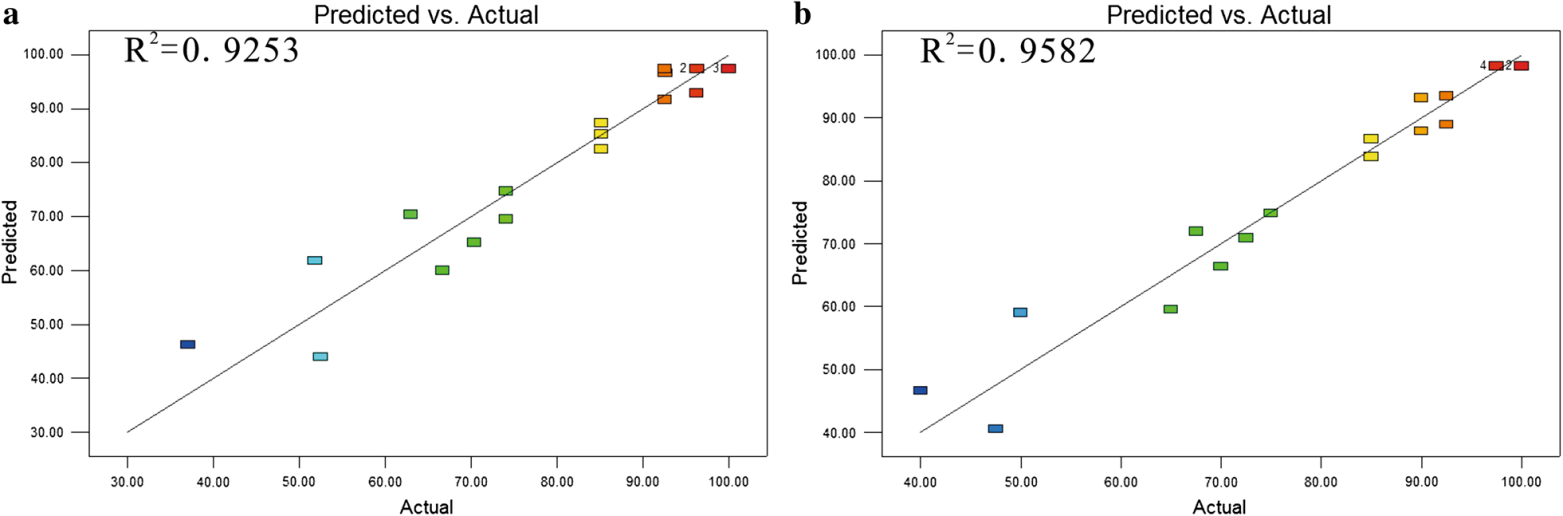
The determination coefficients (R2) between predicted and experimental values: **a** R2 of mode Y1, **b** R2 of mode Y2

**Fig. 3:**
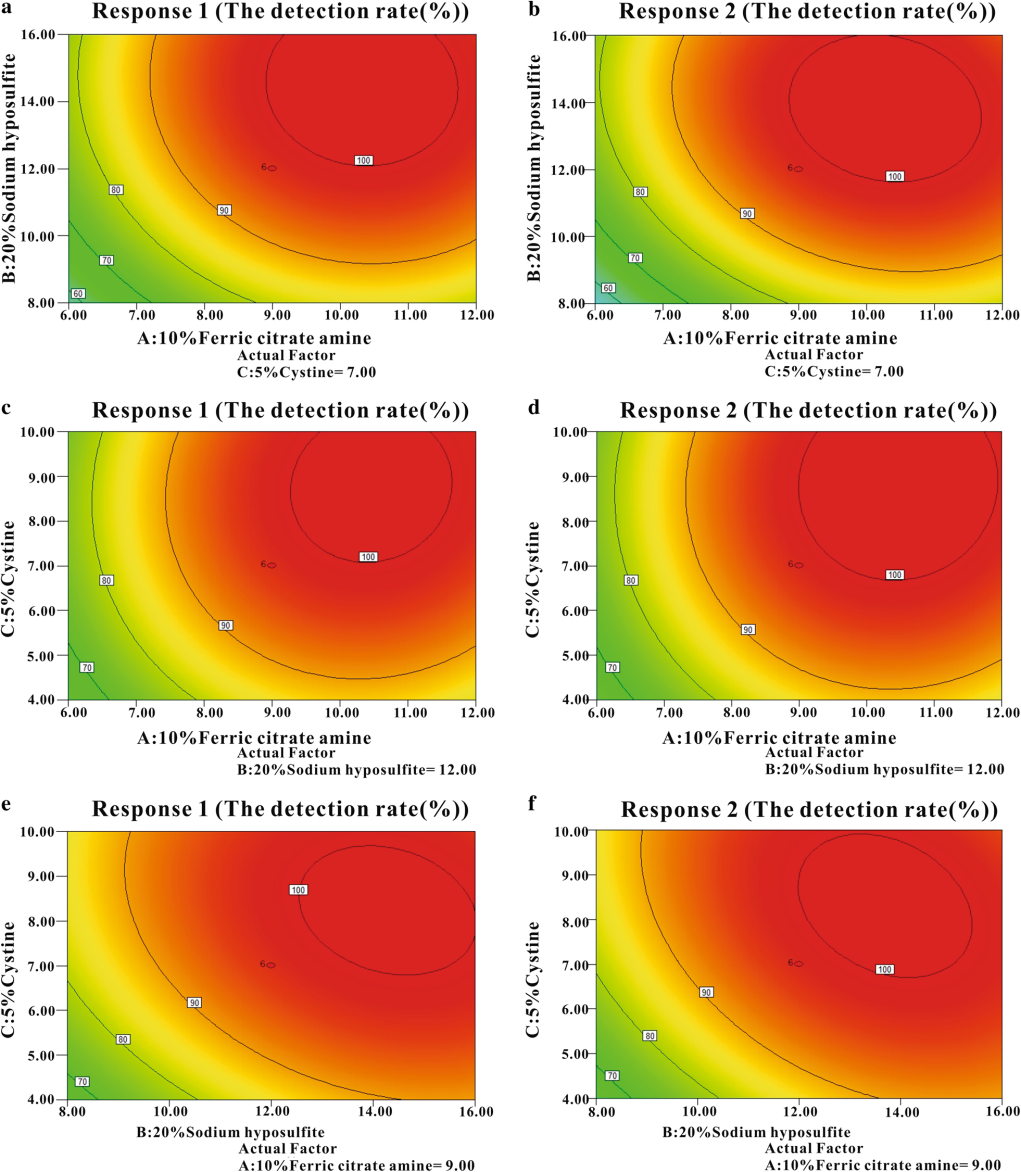
Contour plots from the models: two-dimensional contour plots were generated to display the interactions of the three variables to the response. These contour plots were generated by keeping one variable at a constant position and assessing the action of the other two variables. In the models, 5% cystine (**a**, **b**), 20% sodium hyposulfite (**c**, **d**) and 10% ferric citrate amine (**e**, **f**) were kept at a fixed level in turn

**Fig. 4:**
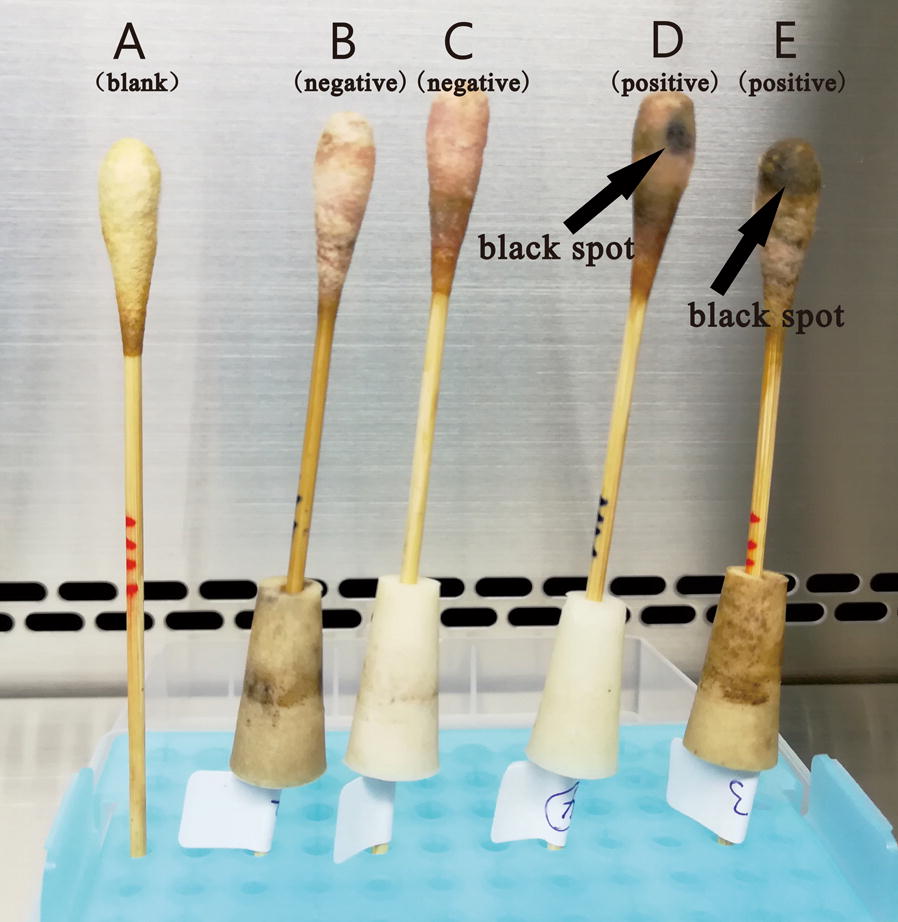
Visual observation of the whole-sample solid-phase enrichment assay using the optimized H_2_S-producing medium: **a** prepared swab with no sample applied, **b**, **c** negative control (swabs with *E. coli* only), **d**, **e** swabs applied with 10^1^ cells of *Salmonella* and 10^5^ cells of *E. coli*

The actual model correlation coefficient was greater than 0.75, which proved that the relevant model has better suitability (Brophy and Joseph 1997). In this study, the determination coefficients (R^2^) (0.9253 for Y1 and 0.9582 for Y2) indicated a good agreement between predicted and experimental values (Fig. 2). ANOVA and the corresponding post hoc contrast were used to evaluate the statistical significance and the goodness-of-fit of two models. The values of the adjusted determination coefficient-squared (Adj R^2^) (0.8580 for Y1 and 0.9206 for Y2) suggested that more than 85% of the variations were due to the three variables present in the models. Both the model F values for each of the response variables (Table 3) and the model P values (Table 4) implied that the models are significant with very low chance (≤ 0.02%) that model F values (11.13 for Y1, 9.10 for Y2) were large owing to noise. Furthermore, the models were all significant and reliable from the low Prob > F value (≤ 0.001 for Y1 and Y2) and the relatively low variation coefficient (CV%) value (8.69 for Y1, 6.56 for Y2) (Table 3). The significance of each term in the models was evaluated by their corresponding P-value. In general, the values less than 0.05 indicated that these terms were significant (Table 4). RSM analysis further revealed that the combination of 9.00 μL of 10% ferric citrate amine, 12.00 μL of 20% sodium hyposulfite, and 7.00 μL of 5% cystine was an optimal combination for both amplification of *Salmonella* and inhibition of *E. coli* (Fig. 3). The related statistical analysis of the model terms by multiple regression was shown in Table 4. The results of visual observation were shown in Fig. 4.

**Table 4:** Statistical analysis of model terms by multiple regression analysis

Term	Y1			Y2		
	CE	SE	P-value	CE	SE	P-value
Intercept	97.35	2.86	0.0002	98.18	2.16	< 0.001
X1	10.77	1.90	0.0002	11.03	1.43	< 0.0001
X2	9.22	1.90	0.0007	8.89	1.43	< 0.0001
X3	6.31	1.90	0.0076	6.37	1.43	< 0.0012
X1X2	− 0.84	2.48	0.7408	− 2.50	1.87	0.2110
X1X3	1.01	2.48	0.6928	0.63	1.87	0.7452
X2X3	− 2.70	2.48	0.3018	− 3.75	1.87	0.0728
${\text{X}}_{1}^{2}$	− 11.67	1.85	< 0.0001	− 11.66	1.39	< 0.0001
${\text{X}}_{2}^{2}$	− 7.09	1.85	0.0033	− 8.57	1.39	0.0001
${\text{X}}_{3}^{2}$	− 5.78	1.85	0.0107	− 5.48	1.39	0.0028

*Y1* mode 1 (the nine type strains), *Y2* mode 2 (the 40 different local strains), *CE* coefficient estimate, *SE* Squares for Error

Through this statistical experiment, we determined the optimal formula containing 0.60% polyvalent poly peptone, 0.40% buffered peptone water, 0.09% ferric citrate amine, 0.24% sodium hyposulfite, 0.035% cystine, 0.01 µg mL^−1^ super absorbing polymer, 0.011% sodium deoxycholate, 15.00 µg mL^−1^ ethyl green and 30.00 µg mL^−1^ sodium selenite. Ethyl green and sodium selenite were used as the bacterial inhibiting agents to improve the specificity of detection. The medium was named as H_2_S-producing medium and used for all the experiments.

### Analytic sensitivity

Our result showed that when the concentration of *E. coli* was 10^4^ cells mL^−1^ or 10^5^ cells mL^−1^, *Salmonella* was detected at a concentration of 10^1^ cells mL^−1^ at 37 °C for 24 h. However, when the concentration of *E. coli* was 10^6^ cells mL^−1^, *Salmonella* (10^1^ cells mL^−1^) was not detectable at 37 °C for 24 h, 36 h or 48 h. Therefore, the analytic sensitivity was determined as 10^1^ cells mL^−1^ with a concentration of interfering bacteria (*E. coli*) at 10^5^ cells mL^−1^ at 37 °C for 24 h.

### Application of WSEA on human feces samples

A total of 4006 samples were collected and synchronously detected for *Salmonella* by the conventional method and the optimized solid phase enrichment. The positive rate was 0.42% by the conventional culture-based method, and 2.12% by WSEA. The proportional positive rate was the highest in the 18th h (50.59%) according to the WSEA. The rate in the 21th h (38.82%) was the second and in the 24 h (9.41%) was the last (Table 5). To improve the detection efficiency, the distinguishment and isolation should be scheduled to be completed in 18–21 h.

**Table 5:** Statistic comparison of traditional method and WSEA on human feces samples

WCDC group	# of samples	Traditional method		Whole-sample solid-phase enrichment assay (WSEA)					
		Positive # (rate)	Positive #/rate	18 h		21 h		24 h	
				Dark spots # (rate)	Positive # (proportion)	Dark spots # (rate)	Positive # (proportion)	Dark spots # (rate)	Positive # (proportion)
1	1502	6 (0.40%)	31 (2.06%)	242 (16.11%)	16 (51.61%)	525 (34.95%)	12 (38.71%)	989 (65.85%)	3 (9.68%)
2	1284	5 (0.39%)	36 (2.8%)	179 (13.94%)	19 (52.78%)	432 (33.64%)	14 (38.89%)	871 (67.83%)	3 (8.33%)
3	1220	6 (0.49%)	18 (1.48%)	123 (10.08%)	9 (50%)	354 (29.02%)	7 (38.89%)	782 (64.10%)	2 (11.11%)
TOTAL	4006	17 (0.42%)	85 (2.12%)	544 (13.58%)	43 (50.59%)	1311 (32.73%)	33 (38.82%)	2642 (65.95%)	8 (9.41%)

## Discussion

On the solid-phase support, the black spots formed by the ferrous sulfide on the swab provide targeted display that helps achieve *in situ* isolation of suspicious colonies. However, inappropriate media formula could lead to false positive or false negative in practical applications. It is crucial to design a universal and efficient WSEA to detect different serotypes of *Salmonella*.

In this study, we found that the amount of hydrogen sulfide produced by reduction of either cystine or sodium thiosulfate is different, depending the type of *Salmonella*. We have found that the proper proportion of cystine and sodium thiosulfate is important in the chromogenic medium of WSEA when detecting various serotypes of *Salmonella*.

As a powerful statistical tool for regression analysis, RSM is specifically applied to study the relationship among multiple factors (Karichappan et al. 2014). Variances had significant effect on regression analysis when adequate precision was greater than 4 (Ramanan et al. 2010). In the regression analysis, the high adequate precision (Adeq P) value (Y1 = 10.77, Y2 = 15.45) indicates that the model is suitable for predicting the responses. The fitness of the models was largely dependent on the determination coefficient (Montgomery 2001). Normally, the value of the determination coefficient (R^2^) ranges from 0 to 1. The closer the value is to 1, the better the model fits the data. Indeed, higher R^2^ (0.9253 for Y1 and 0.9582 for Y2) indicated a good agreement between predicted and experimental values.

In RSM analysis, the two-dimensional contour plots (Fig. 3) were the graphical representations of the regression models and clearly spread the type and significance of interactions between the variances on the responses. The elliptical or saddle contour plot showed the interaction significant (Montgomery 2001). The interaction between B (sodium hyposulfite) and C (cystine) had the highest effect on *Salmonella*; the interaction between A (ferric citrate amine) and B (sodium hyposulfite) was the second, and the interaction between A (ferric citrate amine) and C (cystine) was the last. The exploration on interactions between the factors by the contour plots was helpful in selecting variable ranges to achieve the best optimization.

We successfully employed RSM to develop a solid phase enrichment medium, which included multiple components that could affect the effectiveness of the medium. The optimized WSEA was tested with 4006 patients’ fecal samples and was proven to be effective, further confirming that RSM may have broader applications on experimental designs in the biomedical and clinical fields. Compared with the traditional culture-based method, the positive detection rate from the solid phase enrichment method was more than five times higher. A critical requirement for WSEA is careful observation between the 18th to 21th h of the culture. When the ferrous sulfide-produced black spots spread, the accuracy of isolation for targeted bacteria will decrease due to the blurred positioning of the colony.

The conventional culture-based method for *Salmonella* detection has three main deficiencies. First, the biggest challenge of the traditional enrichment method is the difficulty in providing adequate buffering and protection for targeted bacteria, which directly affects effective recovery and expansion. Under the cyclical antagonism of all non-targeted bacteria, damaged bacteria cannot compete with non-targeted bacteria on the order of magnitude. Second, the traditional method only transfers a loop amount (about 5 μL) from the “pre-enrichment liquid” into the enrichment liquid or the agar plate, which cannot achieve the utilization of a full sample. Third, the low efficiency of traditional detection methods seriously restricts the effective monitoring of public health; it takes 3 to 5 days to obtain any positive or negative results (ICMSF 2011; Velusamy et al. 2010). Due to these constraints, much effort has been devoted to the development of rapid detection technologies, including the enzyme-linked immunosorbent assay (Mirhosseini et al. 2017), polymerase chain reaction (Zhang et al. 2018), and DNA hybridization (Carloni et al. 2018). However, these methods normally require specific immunological or genetic markers, and the diagnosis is often uncertain due to the lack of living bacteria (Tang et al. 2016).

The solid phase enrichment method overcomes many of the drawbacks indicated above. It is capable of adsorbing medium and sample. The selective and chromogenic medium used on this swab can achieve sensitive amplification of target bacteria and form chromogenic colonies *in situ* based on a biochemical reaction. Using naturally degreased cellulose with good biocompatibility (Langer and Tirrell 2004), the fibrous network on the cellulose-based swab provides the site for cell attaching, amplification, and colony formation *in situ*. Moreover, the culture space in swab is divided into several regions with the semi-mobile phase, which greatly reduces the antagonism of non-targeted bacteria in the sample. It also provides sufficient buffer and protection for the trace or damaged target bacteria. In our current study, we showed that cellulose-based swabs are not only effective in absorbing medium for the chromogenic reaction but are also conducive to the antagonism against non-targeted bacteria, possibly due to their large sizes.

Compared to the previously developed immunosensor technique, the WSEA developed in this study also has some advantages (Tang et al. 2016). First, the enrichment medium was optimized using the response surface methodology, an excellent statistical tool for regression analysis. The optimized medium is broadly applicable to a variety of *Salmonella* serotypes. Second, the supplementary inhibiting ingredients were determined as ethyl green 15 μg mL^−1^ and sodium selenite 30 μg mL^−1^. The inhibiting ingredients significantly improved the specificity of *Salmonella* detection. Third, the absorption capacity of the swab was enlarged from 600 to 1000 μL, so that bacteria in the sample were dispersed more widely, which is more conducive to the antagonism of trace *Salmonella* against local non-targeted bacteria. In addition, it is difficult to use the immunosensor technique for large-scale detection. It requires expensive instrument, such as laser scanning confocal microscope, and difficult to prepare the specialized bio-probe reagent at large scale. Through this study, the optimized formulation on solid phase enrichment by RSM allows relatively quick, low-cost, and large-scale detection of *Salmonella*, which could be used in many developing countries. With the role of RSM, We anticipate that commercial products based on this solid phase enrichment detection method for foodborne pathogens will be available soon.

## Additional files

**Additional file 1.** Raw operational data in the central composite design (CCD) in response surface methodology (RSM).

**Additional file 2.** The predicted response for the nine standard *Salmonella* strains (Y1).

**Additional file 3.** The predicted response for the forty *Salmonella* strains from local patients (Y2).

## Data Availability

The data supporting the conclusions are presented in the main article and additional material.

## Abbreviations

WSEA: whole-sample solid-phase enrichment assay
H_2_S: hydrogen sulfide
RSM: response surface methodology
XLD: Xylose Lysine Deoxycholate
MSRV: modified semi-solid rappaport vassiliadis
CCD: central composite design
ANOVA: analysis of variance
ICMSF: International Commission of Microbiological Specializations on Food
