# Supplementary material for: Optimization of an efficient solid-phase enrichment medium for Salmonella detection using response surface methodology
Source: AMB Express. 2019 Jun 28;9:97. doi: 10.1186/s13568-019-0819-0 (PMC6598893; doi:10.1186/s13568-019-0819-0)
Supplement: Supplementary file 1 — Additional file 1. Raw operational data in the central composite design (CCD) in response surface methodology (RSM). [file 13568_2019_819_MOESM1_ESM.pdf]

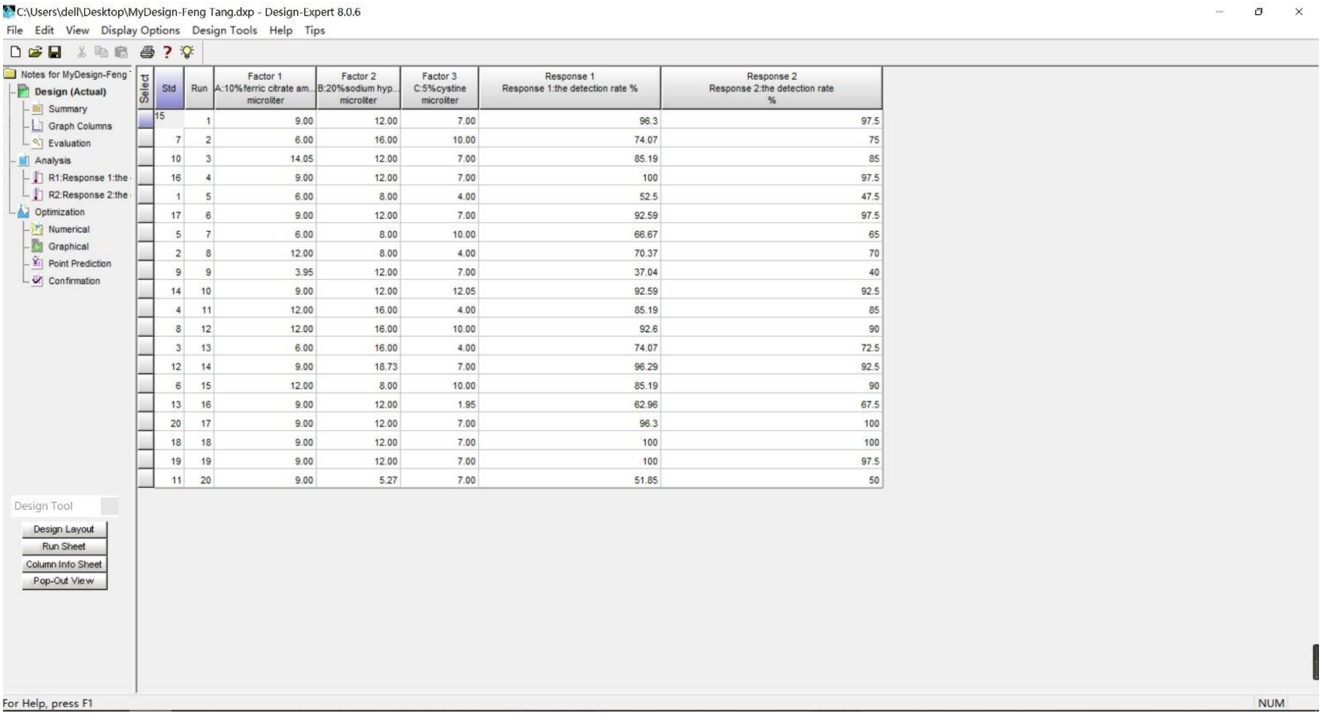

Std	Run	Factor 1 A:10%ferric citrate am... microliter	Factor 2 B:20%sodium hyp... microliter	Factor 3 C:5%cystine microliter	Response 1 Response 1:the detection rate %	Response 2 Response 2:the detection rate %
15	1	9.00	12.00	7.00	96.3	97.5
7	2	6.00	16.00	10.00	74.07	75
10	3	14.05	12.00	7.00	85.19	85
16	4	9.00	12.00	7.00	100	97.5
1	5	6.00	8.00	4.00	52.5	47.5
17	6	9.00	12.00	7.00	92.59	97.5
5	7	6.00	8.00	10.00	66.67	65
2	8	12.00	8.00	4.00	70.37	70
9	9	3.95	12.00	7.00	37.04	40
14	10	9.00	12.00	12.05	92.59	92.5
4	11	12.00	16.00	4.00	85.19	85
8	12	12.00	16.00	10.00	92.6	90
3	13	6.00	16.00	4.00	74.07	72.5
12	14	9.00	18.73	7.00	96.29	92.5
6	15	12.00	8.00	10.00	85.19	90
13	16	9.00	12.00	1.95	62.96	67.5
20	17	9.00	12.00	7.00	96.3	100
18	18	9.00	12.00	7.00	100	100
19	19	9.00	12.00	7.00	100	97.5
11	20	9.00	5.27	7.00	51.85	50

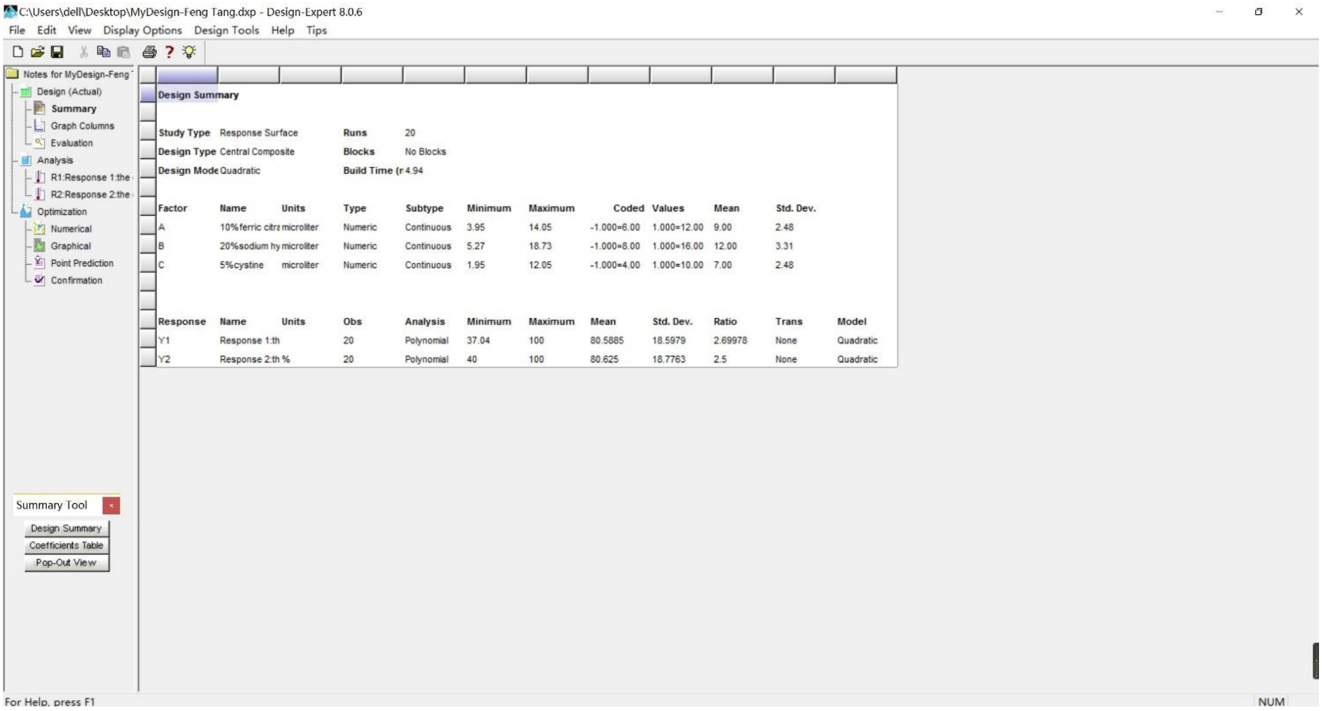

Design Summary

**Study Type**	Response Surface	**Runs**	20
**Design Type**	Central Composite	**Blocks**	No Blocks
**Design Mode**	Quadratic	**Build Time (r**	4.94

Factor	Name	Units	Type	Subtype	Minimum	Maximum	Coded	Values	Mean	Std. Dev.
A	10%ferric citra	microliter	Numeric	Continuous	3.95	14.05	-1.000=6.00	1.000=12.00	9.00	2.48
B	20%sodium hy	microliter	Numeric	Continuous	5.27	18.73	-1.000=8.00	1.000=16.00	12.00	3.31
C	5%cystine	microliter	Numeric	Continuous	1.95	12.05	-1.000=4.00	1.000=10.00	7.00	2.48

Response	Name	Units	Obs	Analysis	Minimum	Maximum	Mean	Std. Dev.	Ratio	Trans	Model
Y1	Response 1:th		20	Polynomial	37.04	100	80.5885	18.5979	2.69978	None	Quadratic
Y2	Response 2:th	%	20	Polynomial	40	100	80.625	18.7763	2.5	None	Quadratic

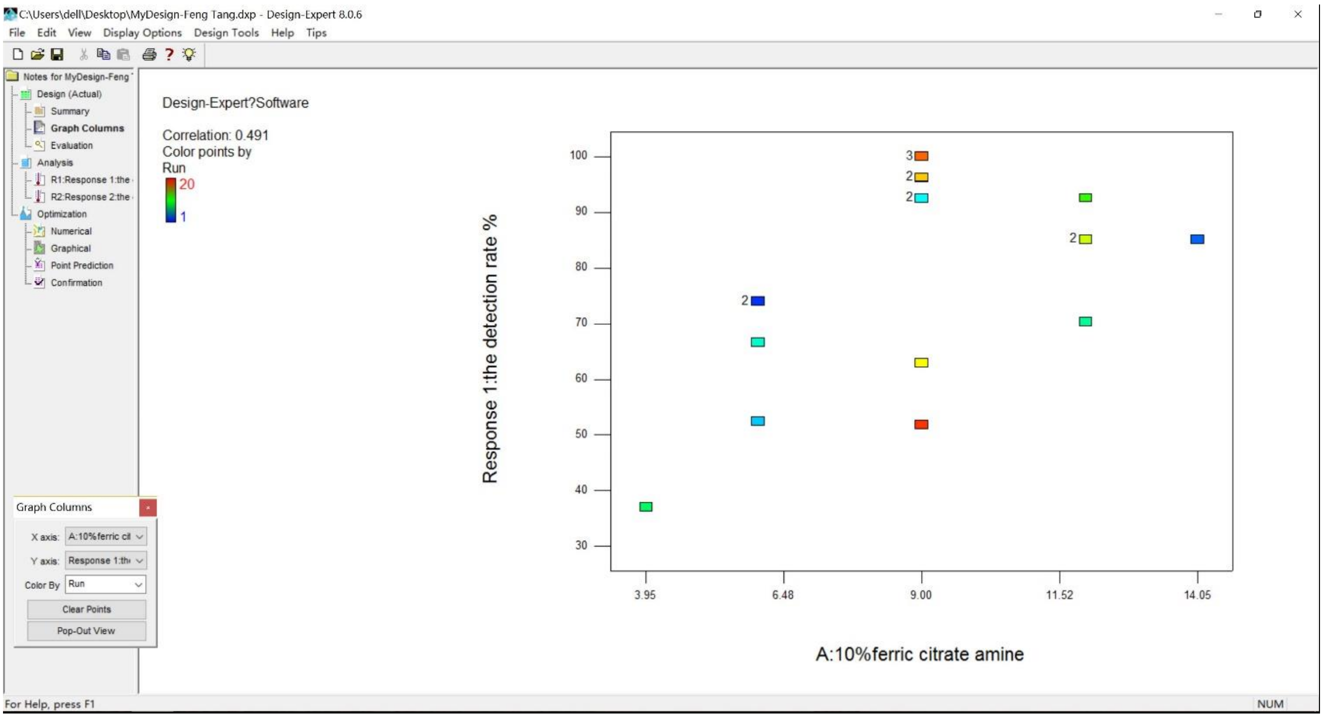
C:\Users\dell\Desktop\MyDesign-Feng Tang.dxp - Design-Expert 8.0.6
File Edit View Display Options Design Tools Help Tips
Design-Expert?Software
Correlation: 0.491
Color points by
Run
20
1
Response 1:the detection rate %
A:10%ferric citrate amine
3.95
6.48
9.00
11.52
14.05
Graph Columns
X axis:
Y axis:
Color By
Clear Points
Pop-Out View
For Help, press F1
NUM

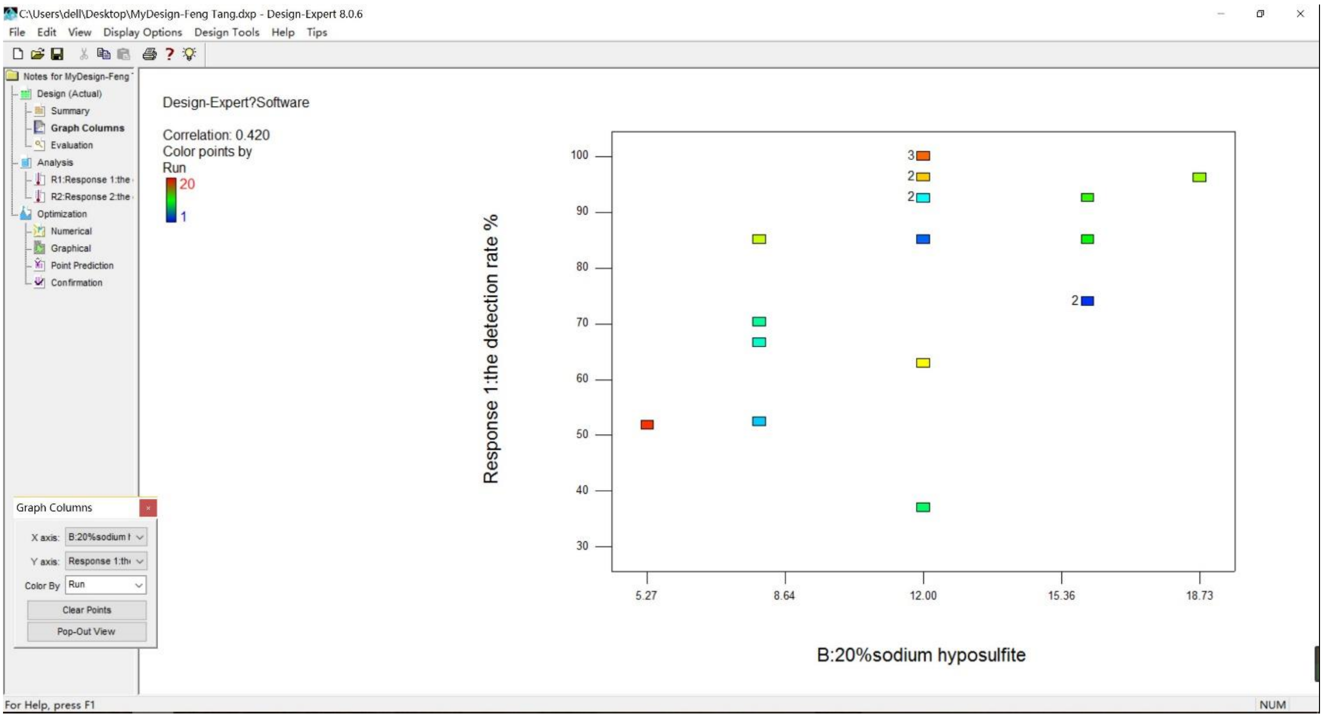
C:\Users\dell\Desktop\MyDesign-Feng Tang.dxp - Design-Expert 8.0.6
File Edit View Display Options Design Tools Help Tips
Design-Expert?Software
Correlation: 0.420
Color points by
Run
20
1
Response 1:the detection rate %
B:20%sodium hyposulfite
5.27
8.64
12.00
15.36
18.73
Graph Columns
X axis:
Y axis:
Color By
Clear Points
Pop-Out View
For Help, press F1
NUM

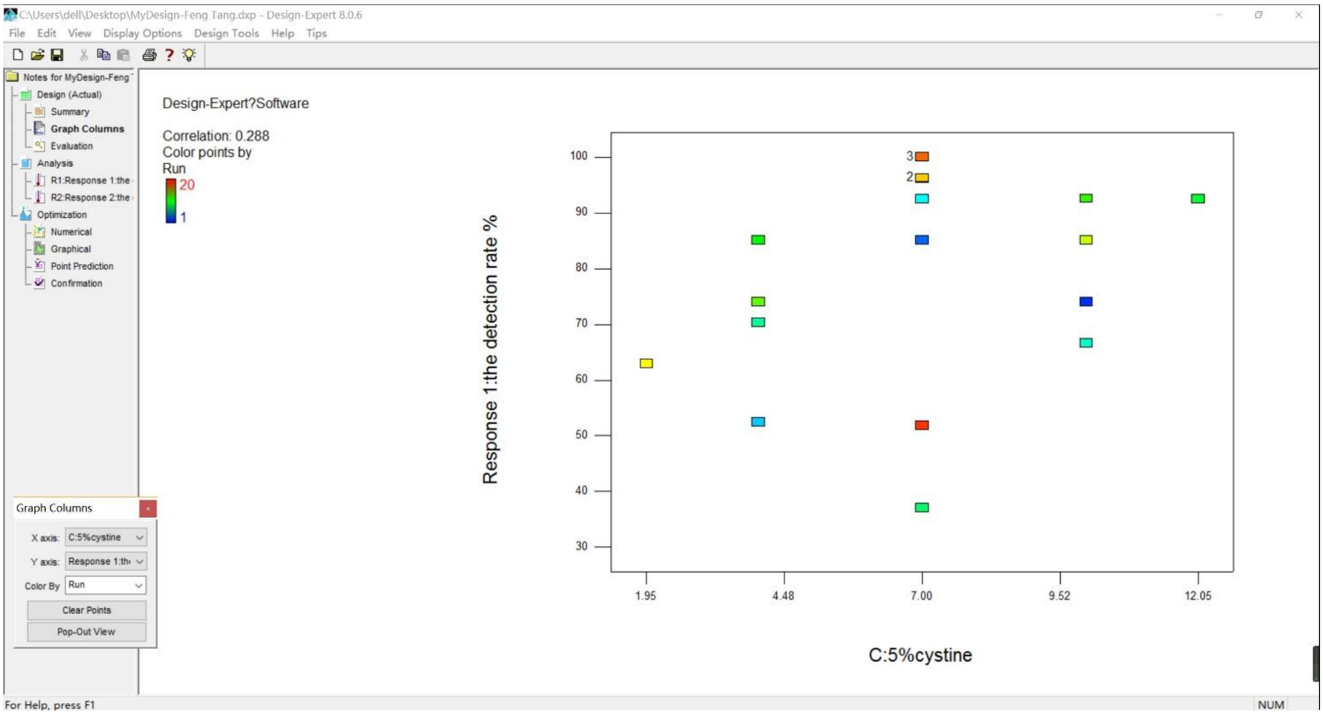
C:\Users\dell\Desktop\MyDesign-Feng Tang.dxp - Design-Expert 8.0.6
File Edit View Display Options Design Tools Help Tips
Notes for MyDesign-Feng
Design (Actual)
Summary
Graph Columns
Evaluation
Analysis
R1:Response 1:the
R2:Response 2:the
Optimization
Numerical
Graphical
Point Prediction
Confirmation
Design-Expert?Software
Correlation: 0.288
Color points by
Run
20
1
Graph Columns
X axis: C:5%cystine
Y axis: Response 1:th
Color By Run
Clear Points
Pop-Out View
Response 1:the detection rate %
C:5%cystine
For Help, press F1
NUM

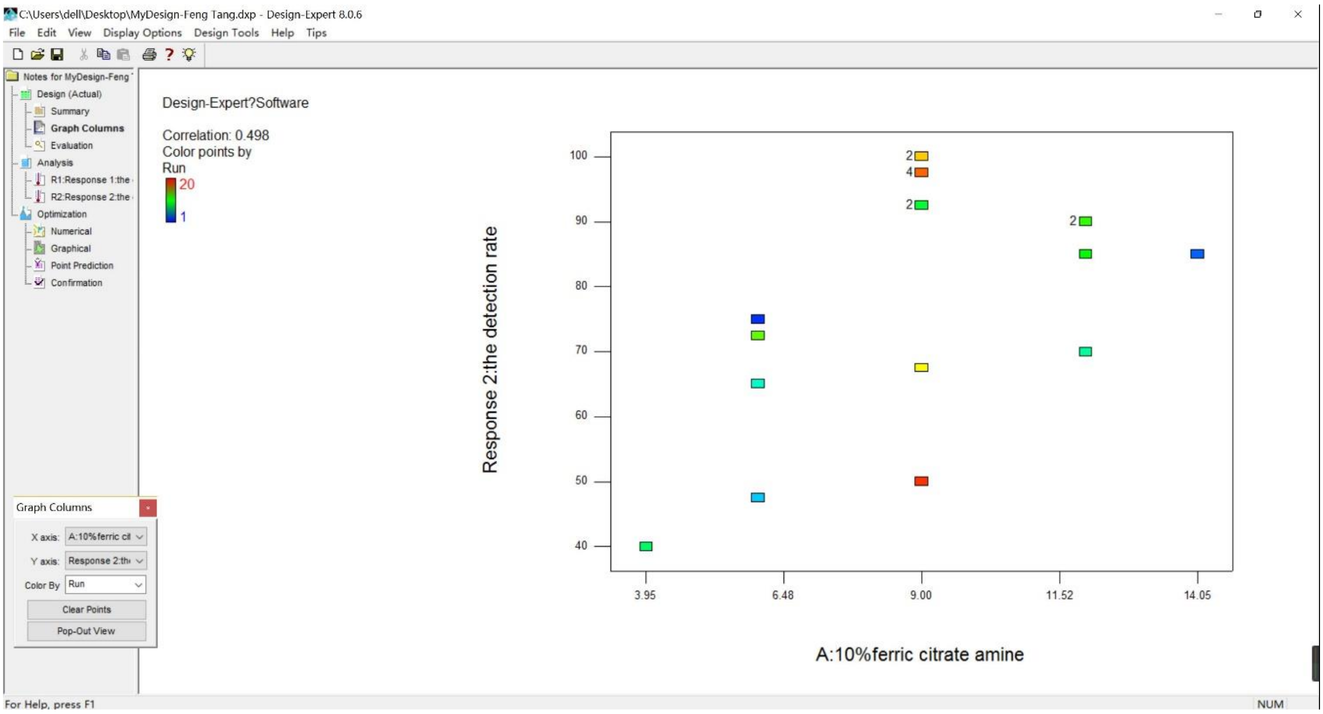
C:\Users\dell\Desktop\MyDesign-Feng Tang.dxp - Design-Expert 8.0.6
File Edit View Display Options Design Tools Help Tips
Design-Expert?Software
Correlation: 0.498
Color points by
Run
20
1
Graph Columns
X axis: A:10%ferric ci
Y axis: Response 2:th
Color By Run
Clear Points
Pop-Out View
Response 2:the detection rate
A:10%ferric citrate amine
For Help, press F1
NUM

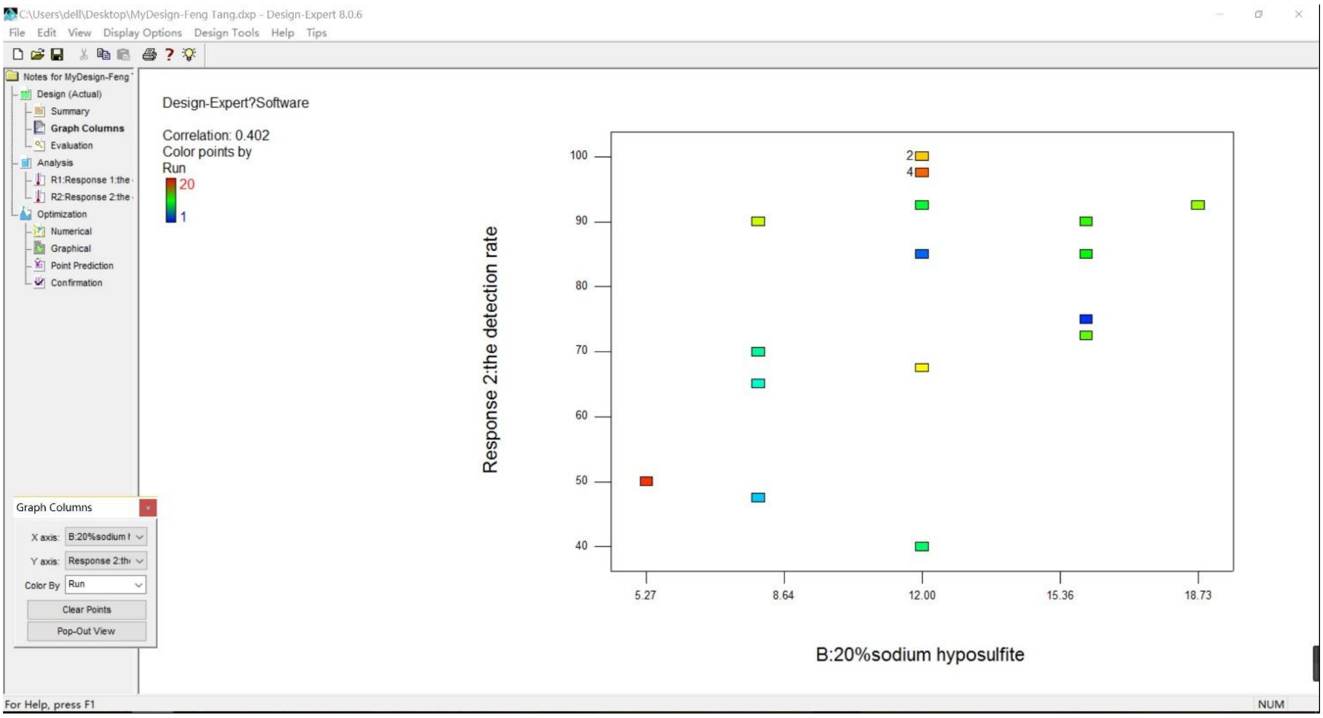
C:\Users\dell\Desktop\MyDesign-Feng Tang.dxp - Design-Expert 8.0.6
File Edit View Display Options Design Tools Help Tips
Notes for MyDesign-Feng
Design (Actual)
Summary
Graph Columns
Evaluation
Analysis
R1:Response 1:the
R2:Response 2:the
Optimization
Numerical
Graphical
Point Prediction
Confirmation
Design-Expert?Software
Correlation: 0.402
Color points by
Run
20
1
Response 2:the detection rate
B:20%sodium hyposulfite
5.27
8.64
12.00
15.36
18.73
100
90
80
70
60
50
40
2
4
Graph Columns
X axis: B:20%sodium h
Y axis: Response 2:th
Color By Run
Clear Points
Pop-Out View
For Help, press F1
NUM

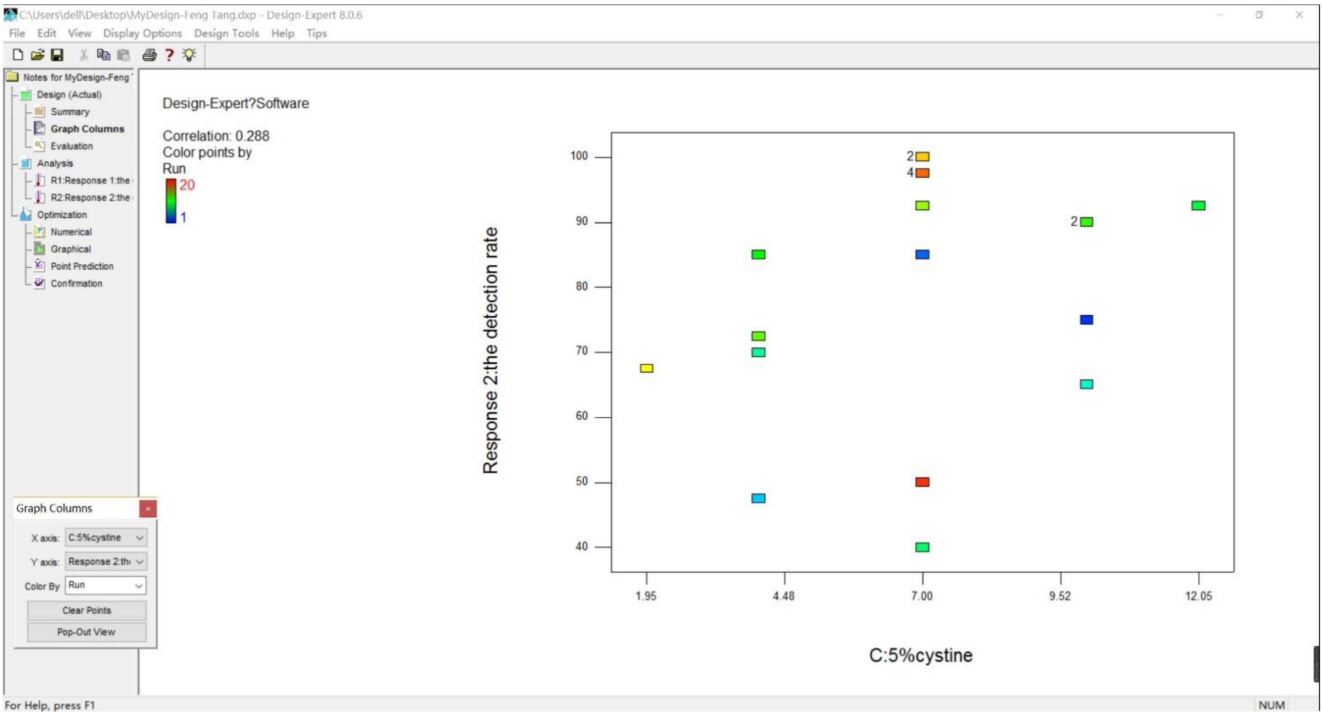
C:\Users\dell\Desktop\MyDesign-Feng Tang.dxp - Design-Expert 8.0.6
File Edit View Display Options Design Tools Help Tips
Notes for MyDesign-Feng
Design (Actual)
Summary
Graph Columns
Evaluation
Analysis
R1:Response 1:the
R2:Response 2:the
Optimization
Numerical
Graphical
Point Prediction
Confirmation
Design-Expert?Software
Correlation: 0.288
Color points by
Run
20
1
Response 2:the detection rate
C:5%cystine
1.95
4.48
7.00
9.52
12.05
100
90
80
70
60
50
40
2
4
2
Graph Columns
X axis: C:5%cystine
Y axis: Response 2:th
Color By Run
Clear Points
Pop-Out View
For Help, press F1
NUM

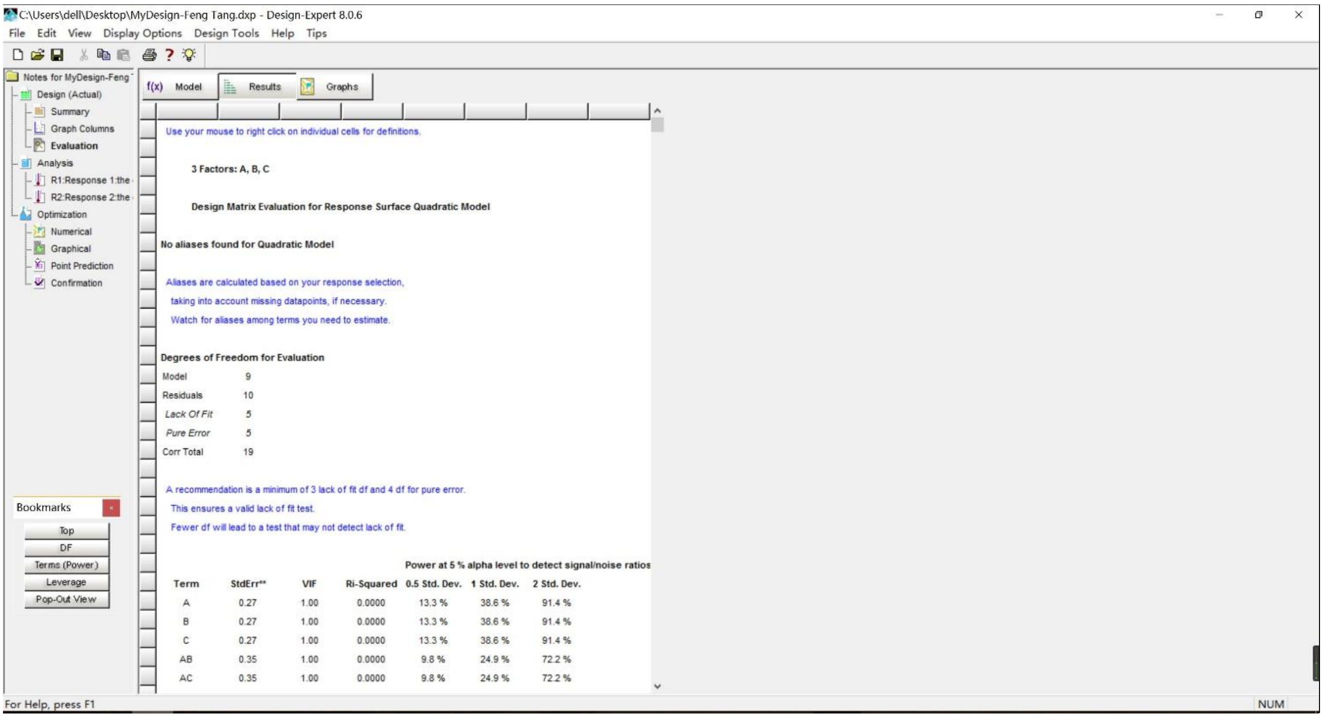

C:\Users\dell\Desktop\MyDesign-Feng Tang.dxp - Design-Expert 8.0.6

Use your mouse to right click on individual cells for definitions.

3 Factors: A, B, C

Design Matrix Evaluation for Response Surface Quadratic Model

No aliases found for Quadratic Model

Aliases are calculated based on your response selection,
taking into account missing datapoints, if necessary.
Watch for aliases among terms you need to estimate.

Degrees of Freedom for Evaluation

Model	9
Residuals	10
Lack Of Fit	5
Pure Error	5
Corr Total	19

A recommendation is a minimum of 3 lack of fit df and 4 df for pure error.
This ensures a valid lack of fit test.
Fewer df will lead to a test that may not detect lack of fit.

				Power at 5 % alpha level to detect signal/noise ratios		
**Term**	**StdErr****	**VIF**	**Ri-Squared**	**0.5 Std. Dev.**	**1 Std. Dev.**	**2 Std. Dev.**
A	0.27	1.00	0.0000	13.3 %	38.6 %	91.4 %
B	0.27	1.00	0.0000	13.3 %	38.6 %	91.4 %
C	0.27	1.00	0.0000	13.3 %	38.6 %	91.4 %
AB	0.35	1.00	0.0000	9.8 %	24.9 %	72.2 %
AC	0.35	1.00	0.0000	9.8 %	24.9 %	72.2 %

For Help, press F1

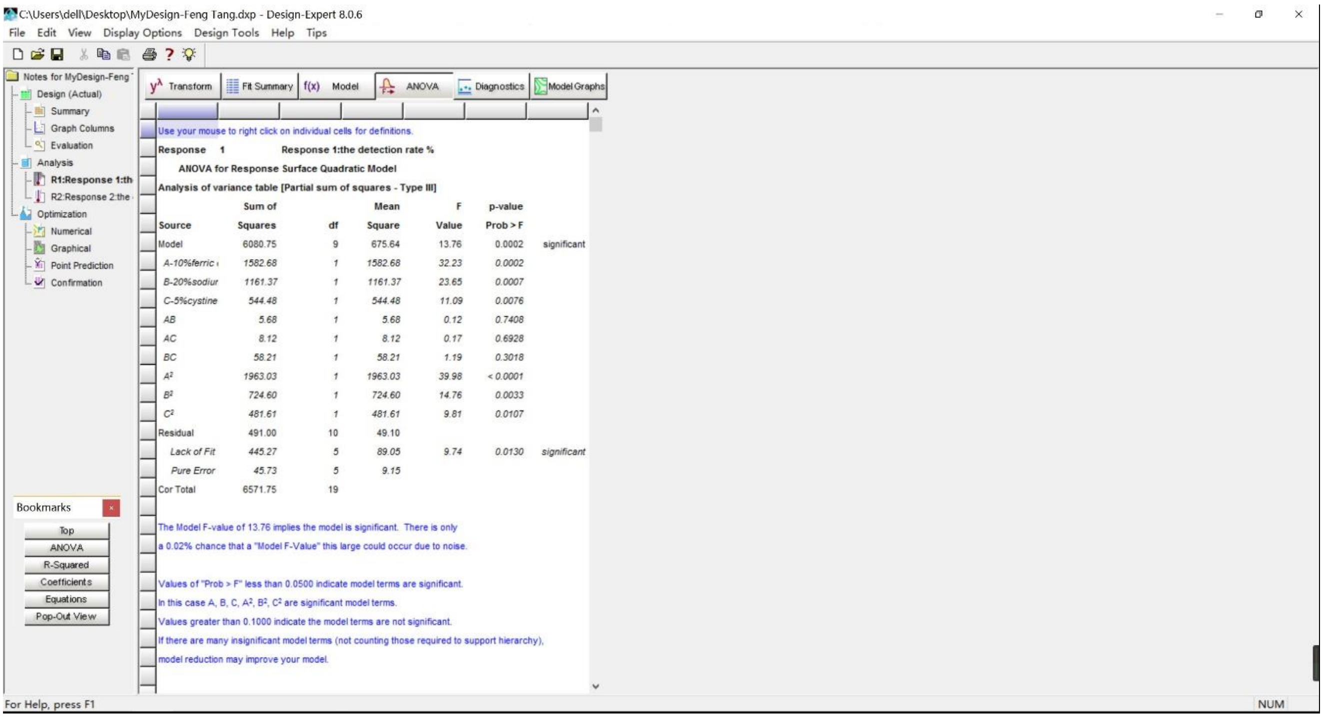

C:\Users\dell\Desktop\MyDesign-Feng Tang.dxp - Design-Expert 8.0.6

Use your mouse to right click on individual cells for definitions.

Response 1 Response 1:the detection rate %

ANOVA for Response Surface Quadratic Model

Analysis of variance table [Partial sum of squares - Type III]

**Source**	**Sum of Squares**	**df**	**Mean Square**	**F Value**	**p-value Prob > F**	
Model	6080.75	9	675.64	13.76	0.0002	significant
A-10%ferric	*1582.68*	*1*	*1582.68*	*32.23*	*0.0002*	
B-20%sodiur	*1161.37*	*1*	*1161.37*	*23.65*	*0.0007*	
C-5%cystine	*544.48*	*1*	*544.48*	*11.09*	*0.0076*	
AB	*5.68*	*1*	*5.68*	*0.12*	*0.7408*	
AC	*8.12*	*1*	*8.12*	*0.17*	*0.6928*	
BC	*58.21*	*1*	*58.21*	*1.19*	*0.3018*	
A^2	*1963.03*	*1*	*1963.03*	*39.98*	*< 0.0001*	
B^2	*724.60*	*1*	*724.60*	*14.76*	*0.0033*	
C^2	*481.61*	*1*	*481.61*	*9.81*	*0.0107*	
Residual	491.00	10	49.10			
Lack of Fit	*445.27*	*5*	*89.05*	*9.74*	*0.0130*	*significant*
Pure Error	*45.73*	*5*	*9.15*			
Cor Total	6571.75	19				

The Model F-value of 13.76 implies the model is significant. There is only
a 0.02% chance that a "Model F-Value" this large could occur due to noise.

Values of "Prob > F" less than 0.0500 indicate model terms are significant.
In this case A, B, C, A^2, B^2, C^2 are significant model terms.
Values greater than 0.1000 indicate the model terms are not significant.
If there are many insignificant model terms (not counting those required to support hierarchy),
model reduction may improve your model.

For Help, press F1

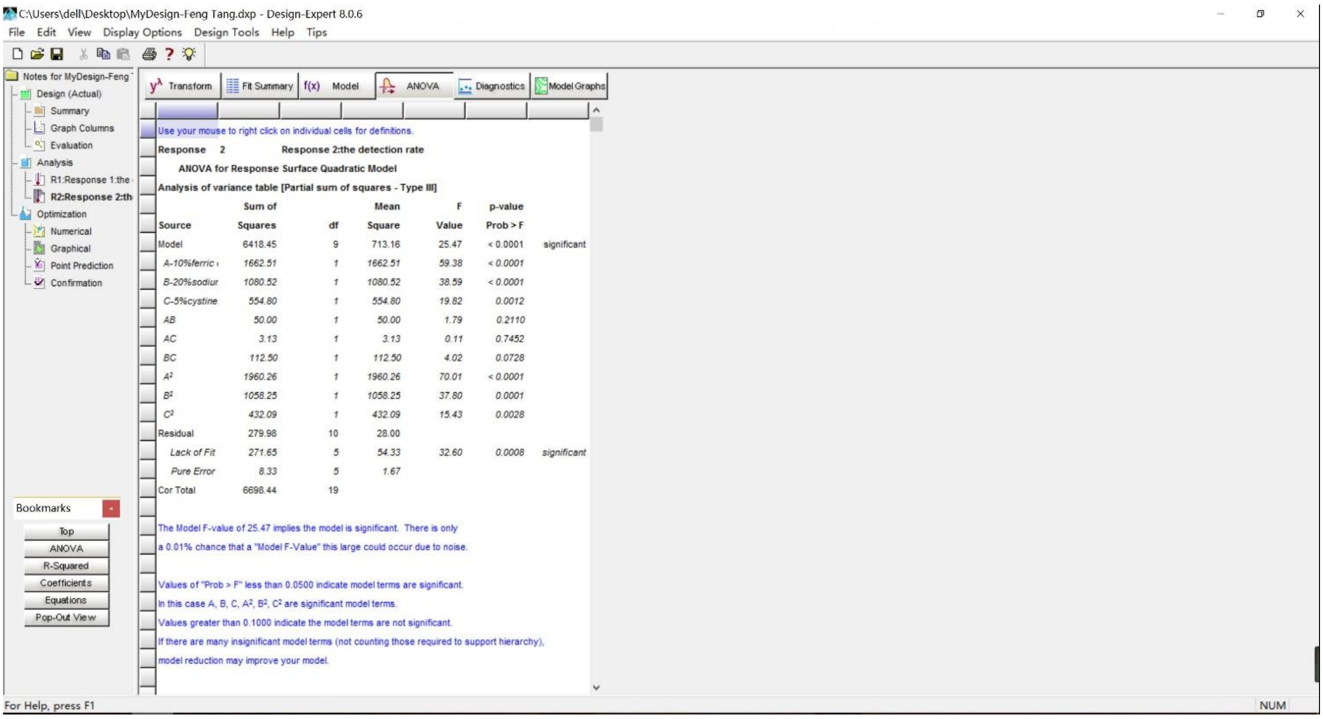

C:\Users\dell\Desktop\MyDesign-Feng Tang.dxp - Design-Expert 8.0.6

Use your mouse to right click on individual cells for definitions.

Response 2 Response 2:the detection rate

ANOVA for Response Surface Quadratic Model

Analysis of variance table [Partial sum of squares - Type III]

Source	Sum of Squares	df	Mean Square	F Value	p-value Prob > F	
Model	6418.45	9	713.16	25.47	< 0.0001	significant
A-10%ferric c	*1662.51*	*1*	*1662.51*	*59.38*	*< 0.0001*	
B-20%sodiur	*1080.52*	*1*	*1080.52*	*38.59*	*< 0.0001*	
C-5%cystine	*554.80*	*1*	*554.80*	*19.82*	*0.0012*	
AB	*50.00*	*1*	*50.00*	*1.79*	*0.2110*	
AC	*3.13*	*1*	*3.13*	*0.11*	*0.7452*	
BC	*112.50*	*1*	*112.50*	*4.02*	*0.0728*	
A^2	*1960.26*	*1*	*1960.26*	*70.01*	*< 0.0001*	
B^2	*1058.25*	*1*	*1058.25*	*37.80*	*0.0001*	
C^2	*432.09*	*1*	*432.09*	*15.43*	*0.0028*	
Residual	279.98	10	28.00			
Lack of Fit	*271.65*	*5*	*54.33*	*32.60*	*0.0008*	*significant*
Pure Error	*8.33*	*5*	*1.67*			
Cor Total	6698.44	19				

The Model F-value of 25.47 implies the model is significant. There is only a 0.01% chance that a "Model F-Value" this large could occur due to noise.

Values of "Prob > F" less than 0.0500 indicate model terms are significant.
In this case A, B, C, A^2, B^2, C^2 are significant model terms.
Values greater than 0.1000 indicate the model terms are not significant.
If there are many insignificant model terms (not counting those required to support hierarchy), model reduction may improve your model.

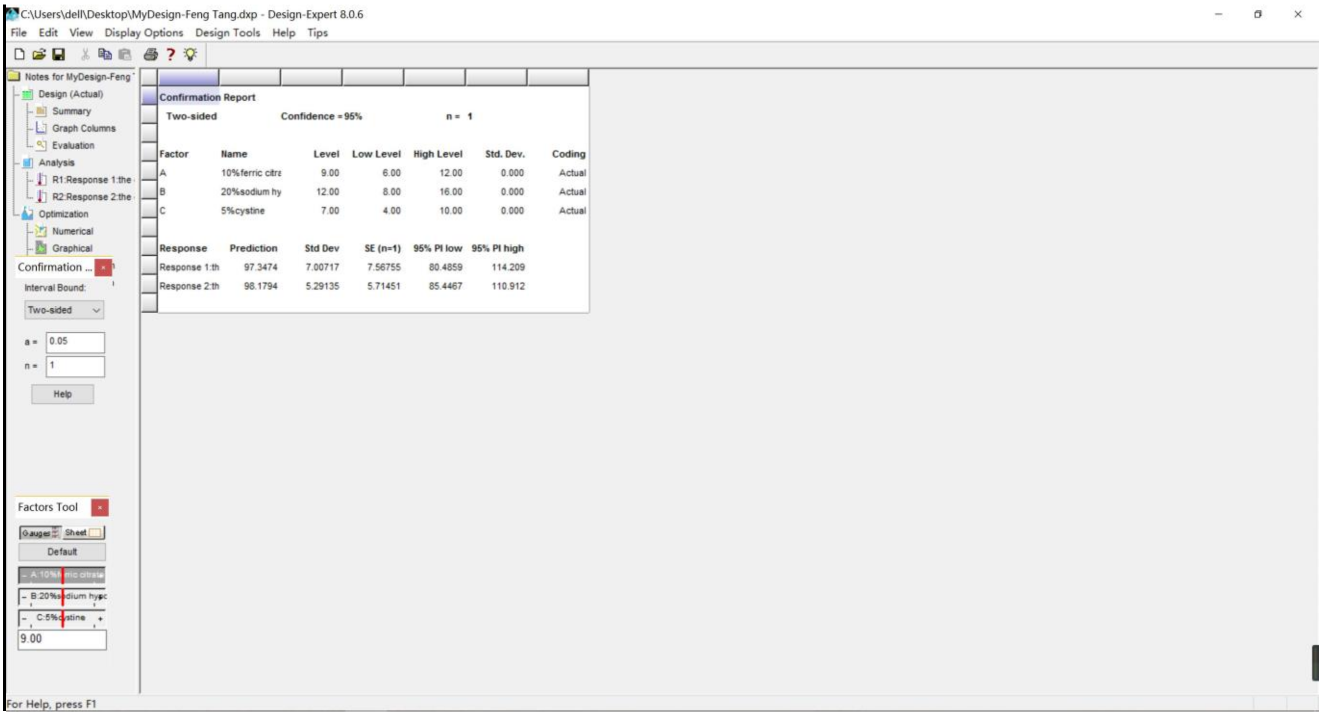

C:\Users\dell\Desktop\MyDesign-Feng Tang.dxp - Design-Expert 8.0.6

Confirmation Report

Two-sided Confidence = 95% n = 1

Factor	Name	Level	Low Level	High Level	Std. Dev.	Coding
A	10%ferric citra	9.00	6.00	12.00	0.000	Actual
B	20%sodium hy	12.00	8.00	16.00	0.000	Actual
C	5%cystine	7.00	4.00	10.00	0.000	Actual

Response	Prediction	Std Dev	SE (n=1)	95% PI low	95% PI high
Response 1:th	97.3474	7.00717	7.56755	80.4859	114.209
Response 2:th	98.1794	5.29135	5.71451	85.4467	110.912

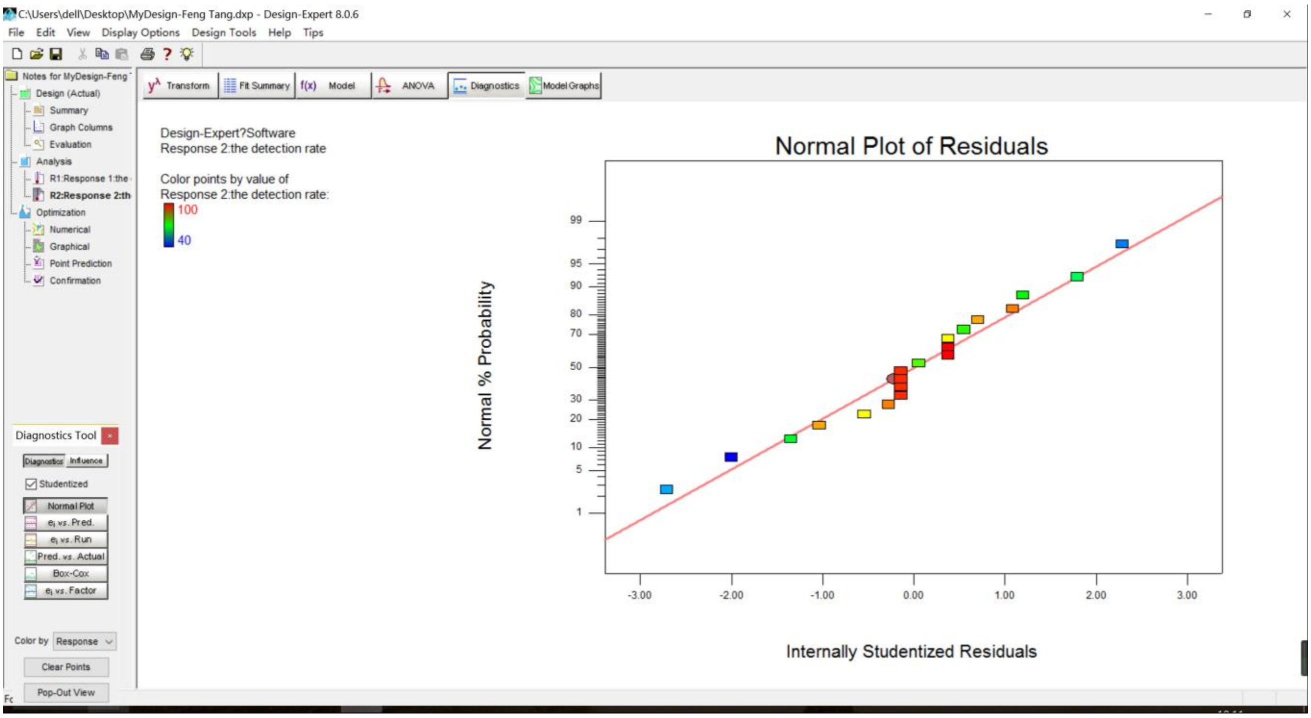
C:\Users\dell\Desktop\MyDesign-Feng Tang.dxp - Design-Expert 8.0.6
File Edit View Display Options Design Tools Help Tips
Notes for MyDesign-Feng
Design (Actual)
Summary
Graph Columns
Evaluation
Analysis
R1:Response 1:the
R2:Response 2:th
Optimization
Numerical
Graphical
Point Prediction
Confirmation
Transform
Fit Summary
f(x) Model
ANOVA
Diagnostics
Model Graphs
Design-Expert?Software
Response 2:the detection rate
Color points by value of
Response 2:the detection rate:
100
40
Normal Plot of Residuals
Normal % Probability
Internally Studentized Residuals
99
95
90
80
70
50
30
20
10
5
1
-3.00
-2.00
-1.00
0.00
1.00
2.00
3.00
Diagnostics Tool
Diagnostics
Influence
Studentized
Normal Plot
Box-Cox
Color by
Response
Clear Points
Pop-Out View

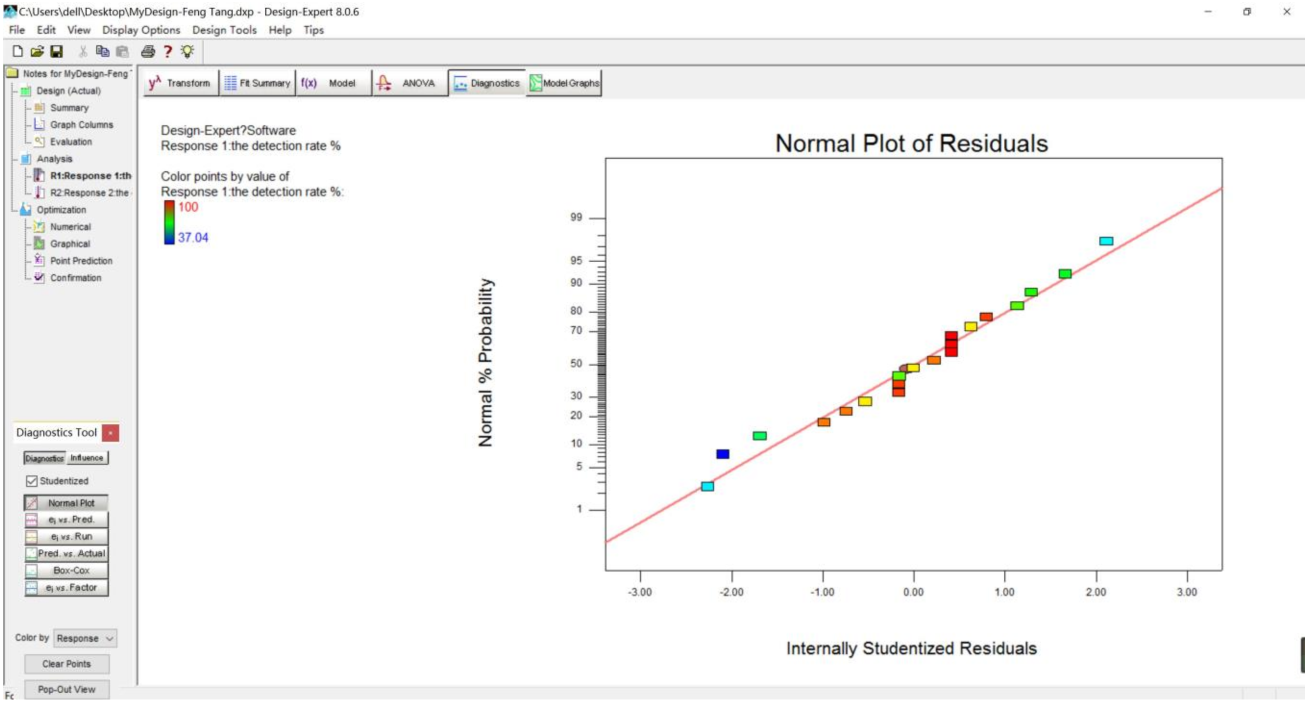
C:\Users\dell\Desktop\MyDesign-Feng Tang.dxp - Design-Expert 8.0.6
File Edit View Display Options Design Tools Help Tips
Notes for MyDesign-Feng
Design (Actual)
Summary
Graph Columns
Evaluation
Analysis
R1:Response 1:th
R2:Response 2:the
Optimization
Numerical
Graphical
Point Prediction
Confirmation
Transform
Fit Summary
f(x) Model
ANOVA
Diagnostics
Model Graphs
Design-Expert?Software
Response 1:the detection rate %
Color points by value of
Response 1:the detection rate %:
100
37.04
Normal Plot of Residuals
Normal % Probability
Internally Studentized Residuals
99
95
90
80
70
50
30
20
10
5
1
-3.00
-2.00
-1.00
0.00
1.00
2.00
3.00
Diagnostics Tool
Diagnostics
Influence
Studentized
Normal Plot
Box-Cox
Color by
Response
Clear Points
Pop-Out View

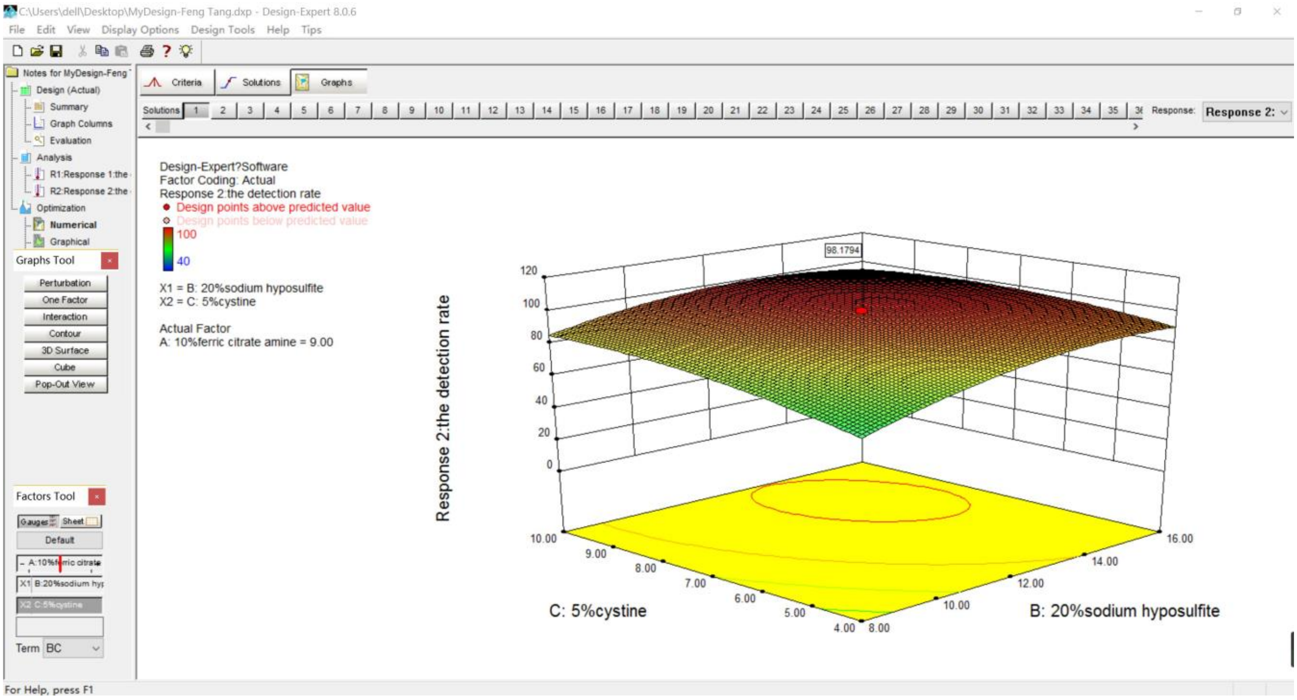
C:\Users\dell\Desktop\MyDesign-Feng Tang.dxp - Design-Expert 8.0.6
Design-Expert?Software
Factor Coding: Actual
Response 2:the detection rate
Design points above predicted value
Design points below predicted value
100
40
X1 = B: 20%sodium hyposulfite
X2 = C: 5%cystine
Actual Factor
A: 10%ferric citrate amine = 9.00
98.1794
Response 2:the detection rate
C: 5%cystine
B: 20%sodium hyposulfite

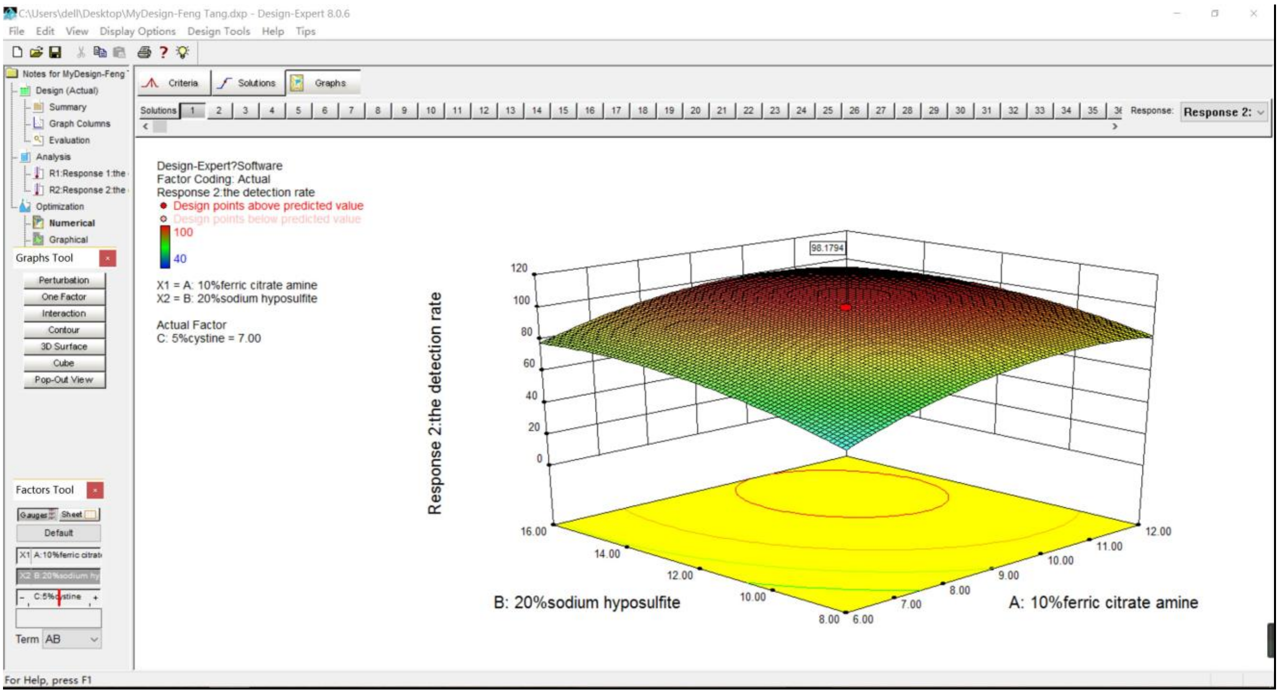
C:\Users\dell\Desktop\MyDesign-Feng Tang.dxp - Design-Expert 8.0.6
Design-Expert?Software
Factor Coding: Actual
Response 2:the detection rate
Design points above predicted value
Design points below predicted value
100
40
X1 = A: 10%ferric citrate amine
X2 = B: 20%sodium hyposulfite
Actual Factor
C: 5%cystine = 7.00
98.1794
Response 2:the detection rate
B: 20%sodium hyposulfite
A: 10%ferric citrate amine

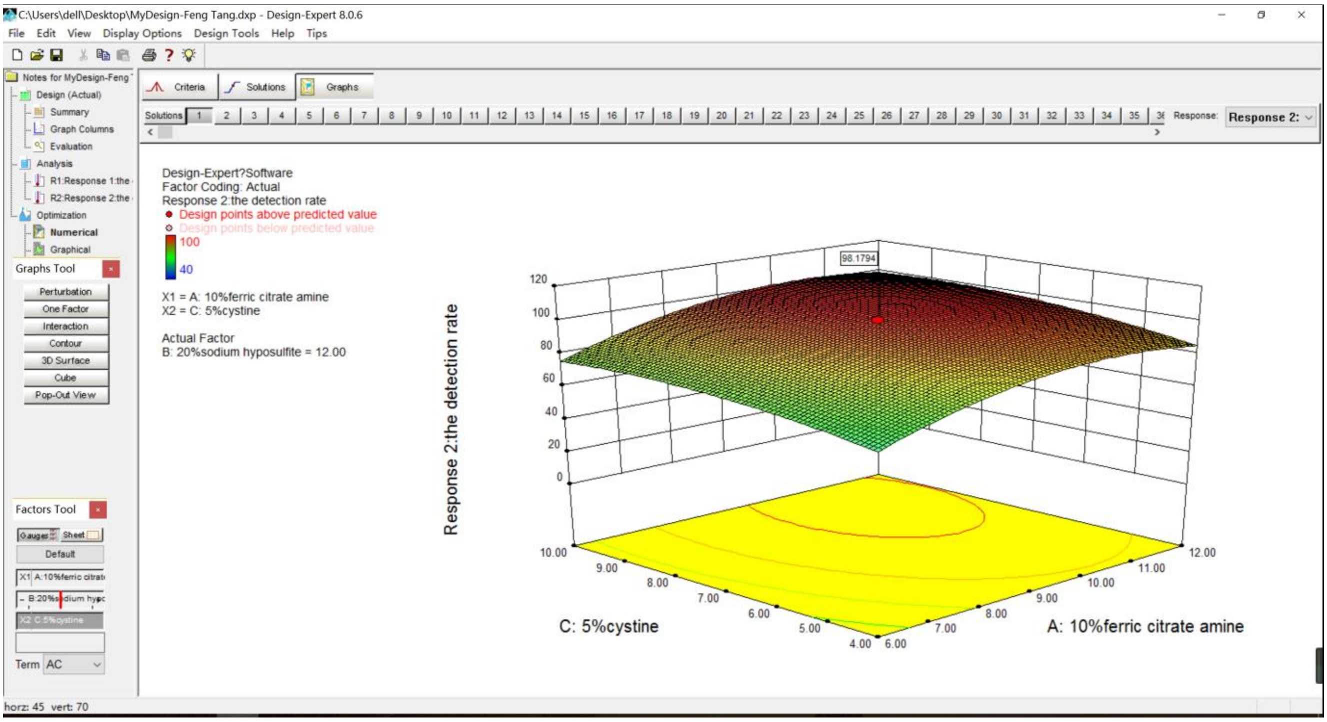
C:\Users\dell\Desktop\MyDesign-Feng Tang.dxp - Design-Expert 8.0.6
File Edit View Display Options Design Tools Help Tips
Criteria
Solutions
Graphs
Response: Response 2:
Notes for MyDesign-Feng
Design (Actual)
Summary
Graph Columns
Evaluation
Analysis
R1:Response 1:the
R2:Response 2:the
Optimization
Numerical
Graphical
Graphs Tool
Perturbation
One Factor
Interaction
Contour
3D Surface
Cube
Pop-Out View
Factors Tool
Default
Term AC
Design-Expert?Software
Factor Coding: Actual
Response 2:the detection rate
Design points above predicted value
Design points below predicted value
100
40
X1 = A: 10%ferric citrate amine
X2 = C: 5%cystine
Actual Factor
B: 20%sodium hyposulfite = 12.00
98.1794
Response 2:the detection rate
C: 5%cystine
A: 10%ferric citrate amine
horz: 45 vert: 70

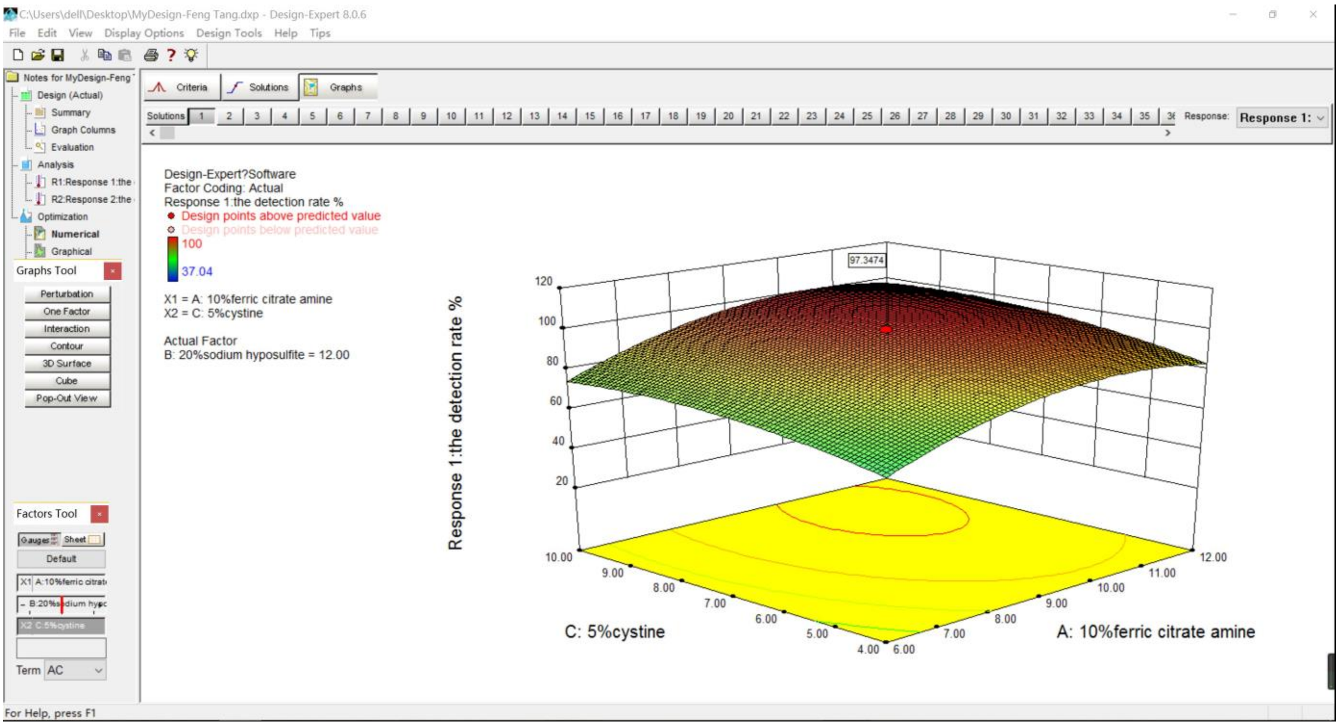
C:\Users\dell\Desktop\MyDesign-Feng Tang.dxp - Design-Expert 8.0.6
File Edit View Display Options Design Tools Help Tips
Criteria
Solutions
Graphs
Response: Response 1:
Notes for MyDesign-Feng
Design (Actual)
Summary
Graph Columns
Evaluation
Analysis
R1:Response 1:the
R2:Response 2:the
Optimization
Numerical
Graphical
Graphs Tool
Perturbation
One Factor
Interaction
Contour
3D Surface
Cube
Pop-Out View
Factors Tool
Default
Term AC
Design-Expert?Software
Factor Coding: Actual
Response 1:the detection rate %
Design points above predicted value
Design points below predicted value
100
37.04
X1 = A: 10%ferric citrate amine
X2 = C: 5%cystine
Actual Factor
B: 20%sodium hyposulfite = 12.00
97.3474
Response 1:the detection rate %
C: 5%cystine
A: 10%ferric citrate amine
For Help, press F1

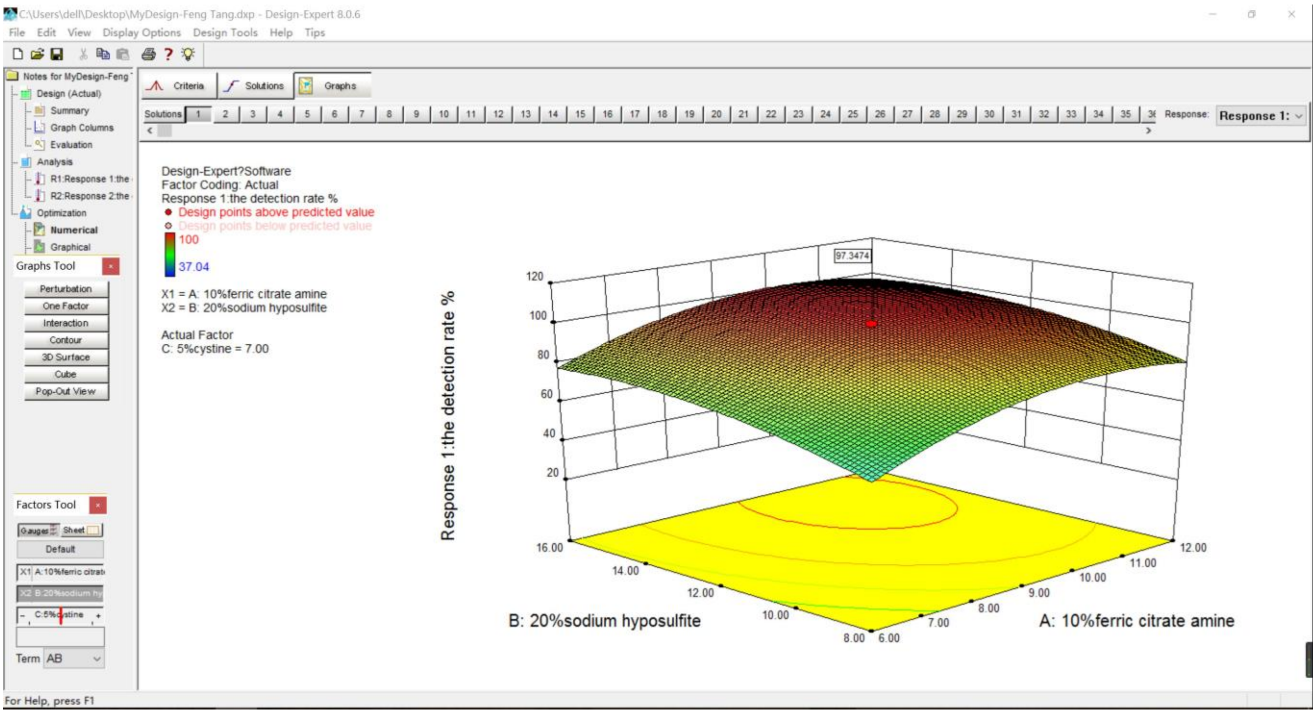
C:\Users\dell\Desktop\MyDesign-Feng Tang.dxp - Design-Expert 8.0.6
File Edit View Display Options Design Tools Help Tips
Criteria
Solutions
Graphs
Response: Response 1:
Design-Expert?Software
Factor Coding: Actual
Response 1:the detection rate %
Design points above predicted value
Design points below predicted value
100
37.04
X1 = A: 10%ferric citrate amine
X2 = B: 20%sodium hyposulfite
Actual Factor
C: 5%cystine = 7.00
97.3474
Response 1:the detection rate %
120
100
80
60
40
20
16.00
14.00
12.00
10.00
8.00
6.00
7.00
8.00
9.00
10.00
11.00
12.00
B: 20%sodium hyposulfite
A: 10%ferric citrate amine
Graphs Tool
Perturbation
One Factor
Interaction
Contour
3D Surface
Cube
Pop-Out View
Factors Tool
Default
Term AB
For Help, press F1

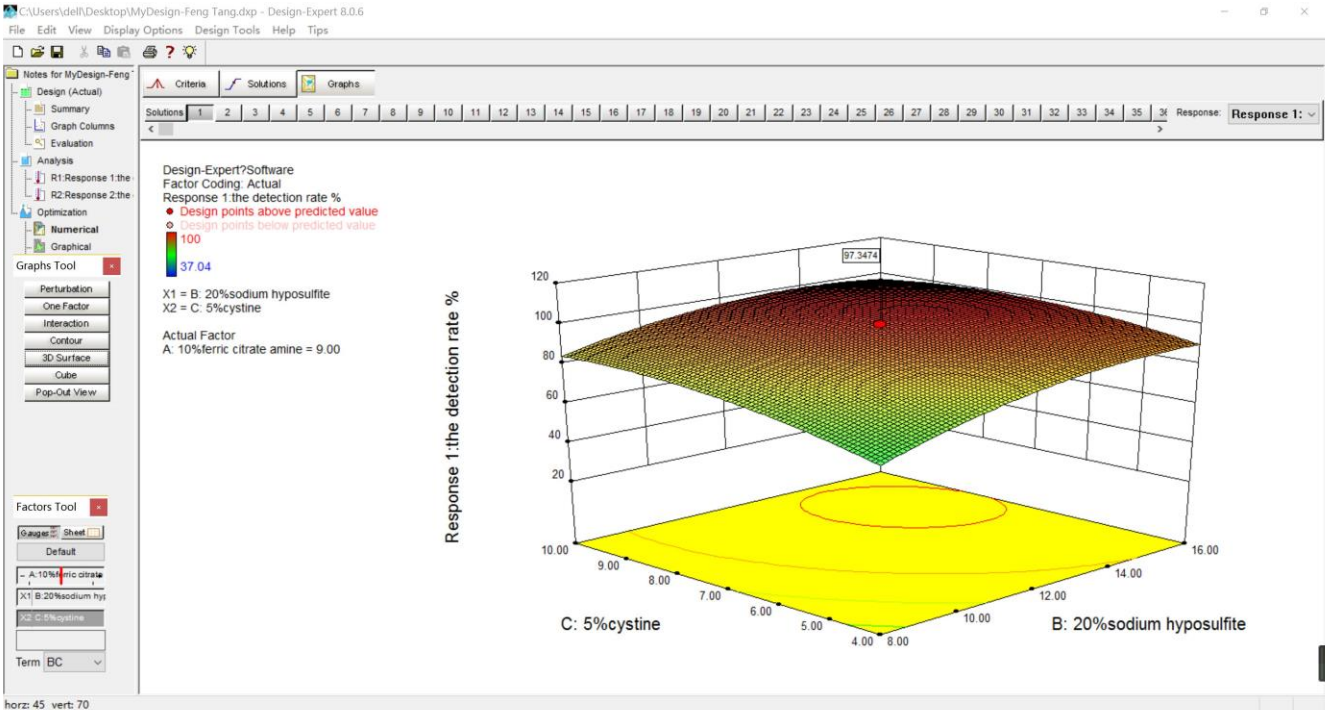
C:\Users\dell\Desktop\MyDesign-Feng Tang.dxp - Design-Expert 8.0.6
File Edit View Display Options Design Tools Help Tips
Criteria
Solutions
Graphs
Response: Response 1:
Design-Expert?Software
Factor Coding: Actual
Response 1:the detection rate %
Design points above predicted value
Design points below predicted value
100
37.04
X1 = B: 20%sodium hyposulfite
X2 = C: 5%cystine
Actual Factor
A: 10%ferric citrate amine = 9.00
97.3474
Response 1:the detection rate %
120
100
80
60
40
20
10.00
9.00
8.00
7.00
6.00
5.00
4.00
8.00
10.00
12.00
14.00
16.00
C: 5%cystine
B: 20%sodium hyposulfite
Graphs Tool
Perturbation
One Factor
Interaction
Contour
3D Surface
Cube
Pop-Out View
Factors Tool
Default
Term BC
horz: 45 vert: 70

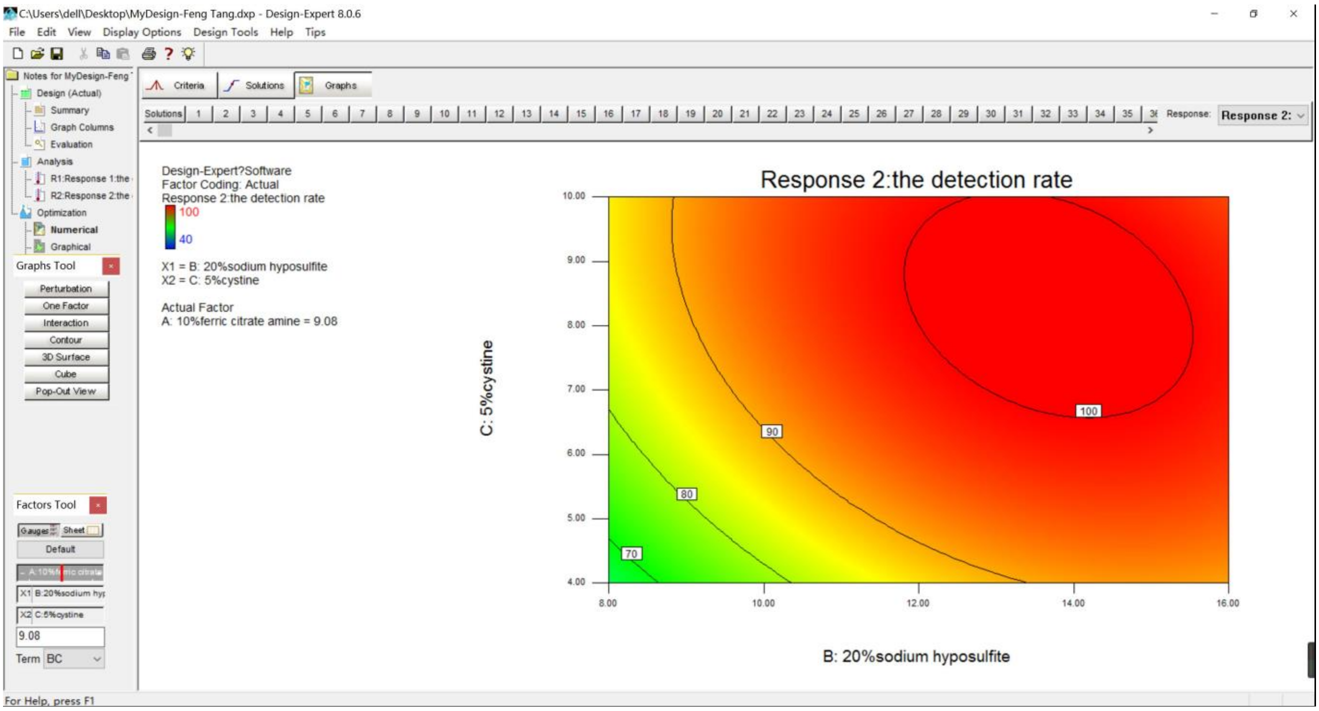
C:\Users\dell\Desktop\MyDesign-Feng Tang.dxp - Design-Expert 8.0.6
Design-Expert?Software
Factor Coding: Actual
Response 2:the detection rate
100
40
X1 = B: 20%sodium hyposulfite
X2 = C: 5%cystine
Actual Factor
A: 10%ferric citrate amine = 9.08
Response 2:the detection rate
C: 5%cystine
B: 20%sodium hyposulfite
70
80
90
100
For Help, press F1

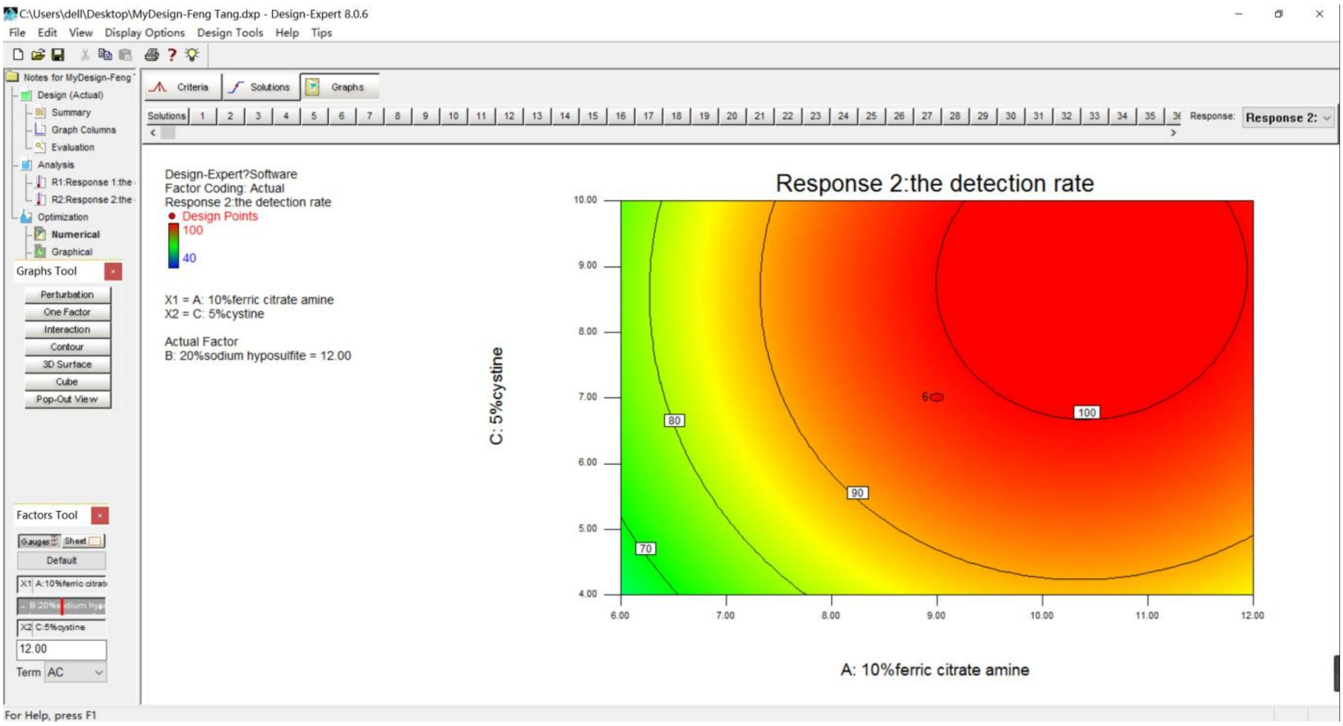
C:\Users\dell\Desktop\MyDesign-Feng Tang.dxp - Design-Expert 8.0.6
Design-Expert?Software
Factor Coding: Actual
Response 2:the detection rate
Design Points
100
40
X1 = A: 10%ferric citrate amine
X2 = C: 5%cystine
Actual Factor
B: 20%sodium hyposulfite = 12.00
Response 2:the detection rate
C: 5%cystine
A: 10%ferric citrate amine
70
80
90
100
For Help, press F1

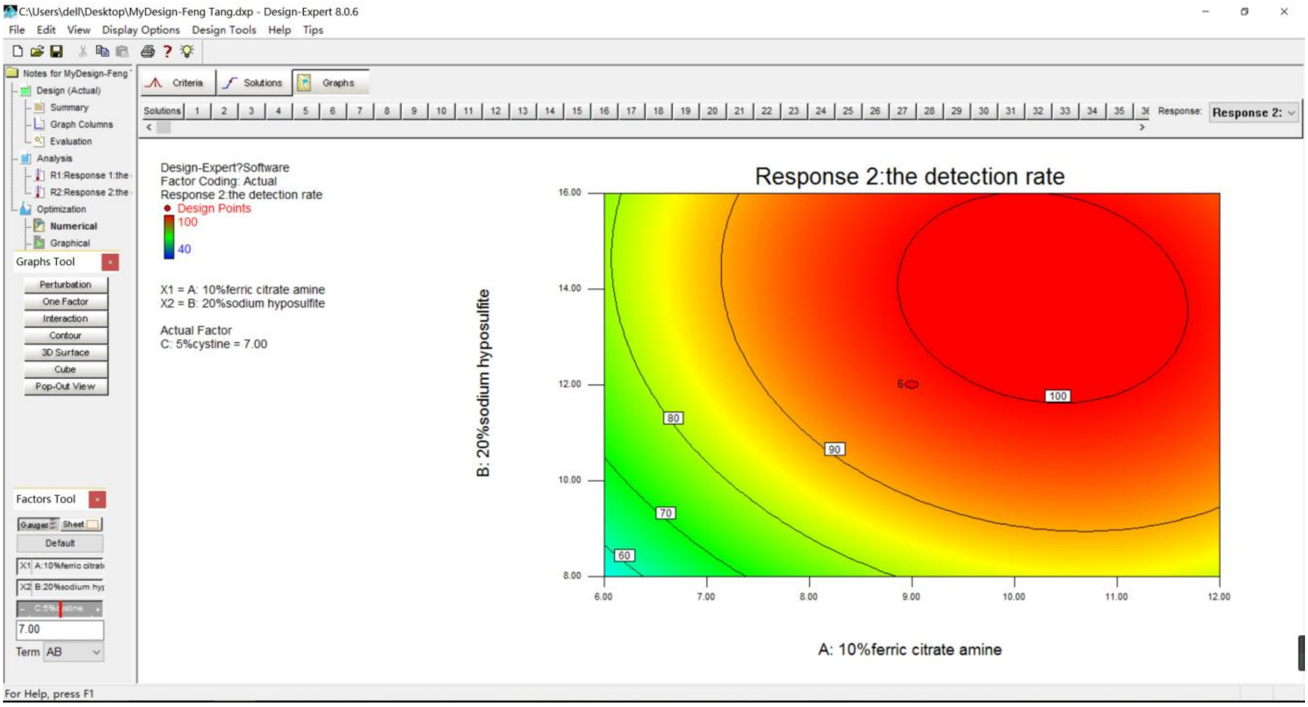
C:\Users\dell\Desktop\MyDesign-Feng Tang.dxp - Design-Expert 8.0.6
Design-Expert?Software
Factor Coding: Actual
Response 2:the detection rate
Design Points
100
40
X1 = A: 10%ferric citrate amine
X2 = B: 20%sodium hyposulfite
Actual Factor
C: 5%cystine = 7.00
Response 2:the detection rate
B: 20%sodium hyposulfite
A: 10%ferric citrate amine
For Help, press F1

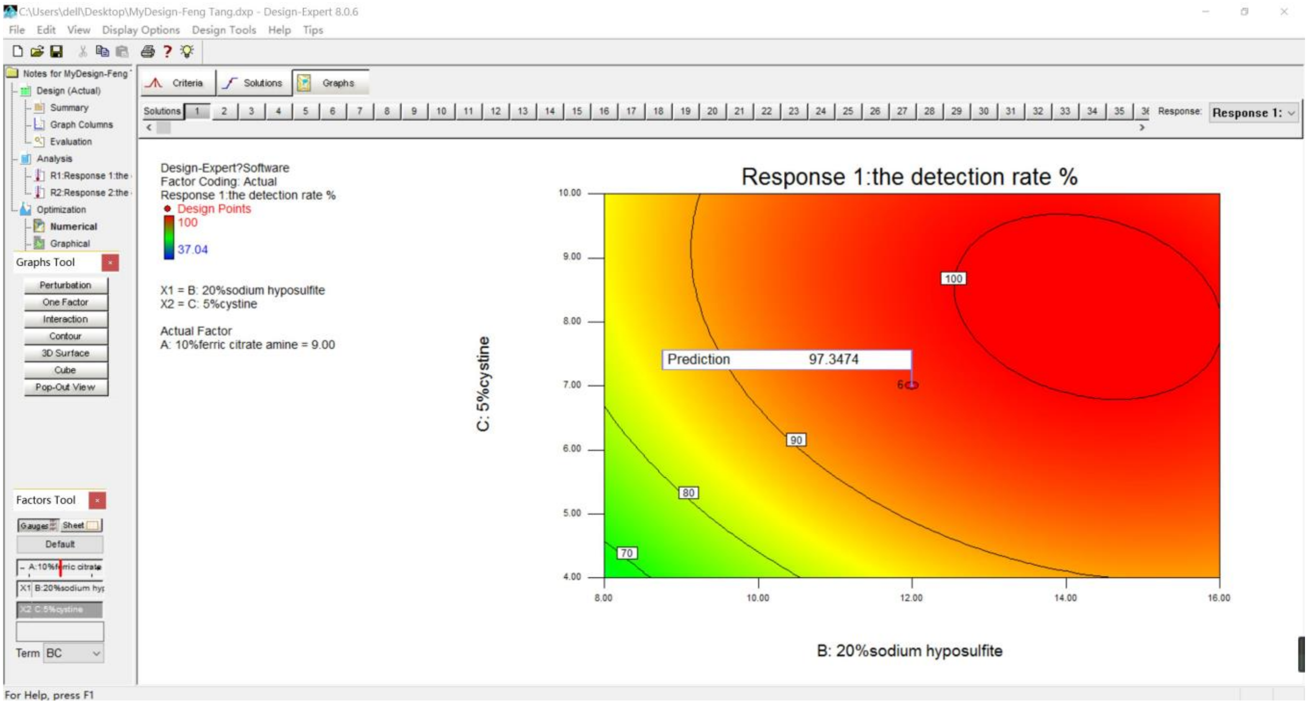
C:\Users\dell\Desktop\MyDesign-Feng Tang.dxp - Design-Expert 8.0.6
Design-Expert?Software
Factor Coding: Actual
Response 1:the detection rate %
Design Points
100
37.04
X1 = B: 20%sodium hyposulfite
X2 = C: 5%cystine
Actual Factor
A: 10%ferric citrate amine = 9.00
Response 1:the detection rate %
Prediction 97.3474
C: 5%cystine
B: 20%sodium hyposulfite
For Help, press F1

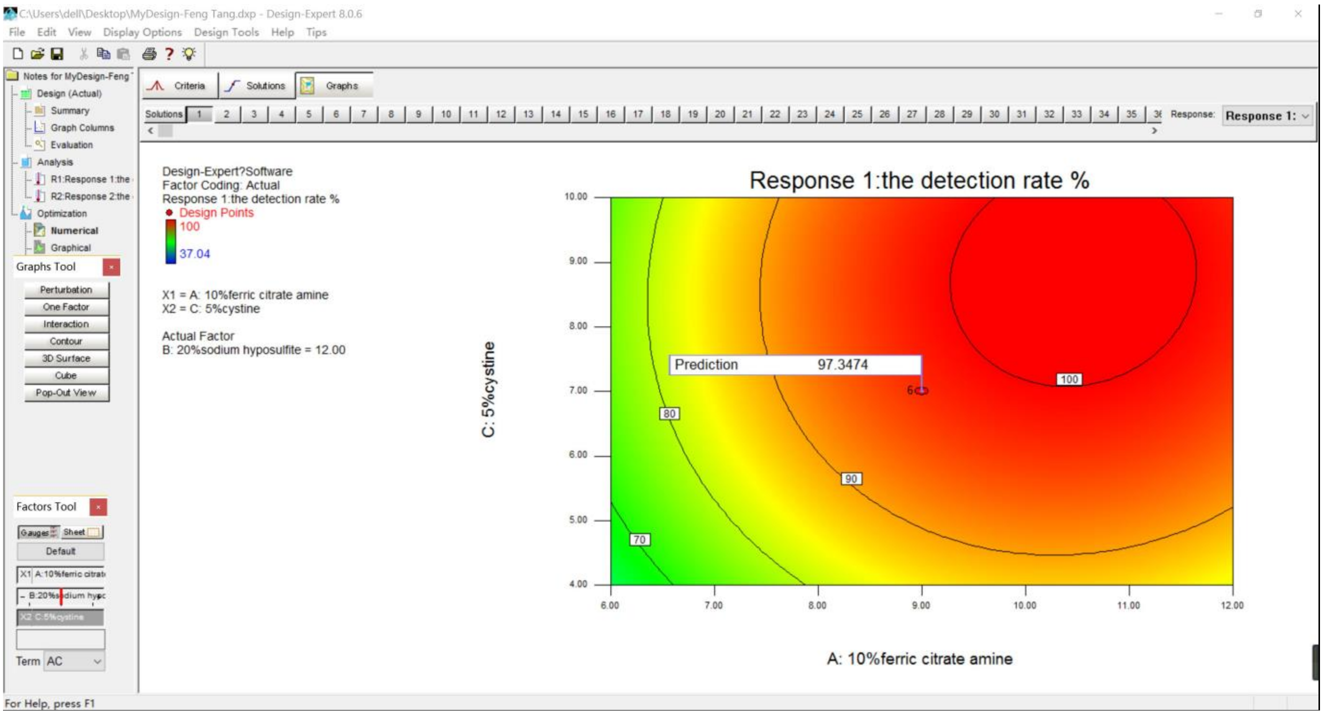
C:\Users\dell\Desktop\MyDesign-Feng Tang.dxp - Design-Expert 8.0.6
File Edit View Display Options Design Tools Help Tips
Criteria
Solutions
Graphs
Response:
Response 1:
Notes for MyDesign-Feng
Design (Actual)
Summary
Graph Columns
Evaluation
Analysis
R1:Response 1:the
R2:Response 2:the
Optimization
Numerical
Graphical
Graphs Tool
Perturbation
One Factor
Interaction
Contour
3D Surface
Cube
Pop-Out View
Factors Tool
Gauges
Sheet
Default
Term AC
Design-Expert?Software
Factor Coding: Actual
Response 1:the detection rate %
Design Points
100
37.04
X1 = A: 10%ferric citrate amine
X2 = C: 5%cystine
Actual Factor
B: 20%sodium hyposulfite = 12.00
Response 1:the detection rate %
Prediction 97.3474
C: 5%cystine
A: 10%ferric citrate amine
For Help, press F1

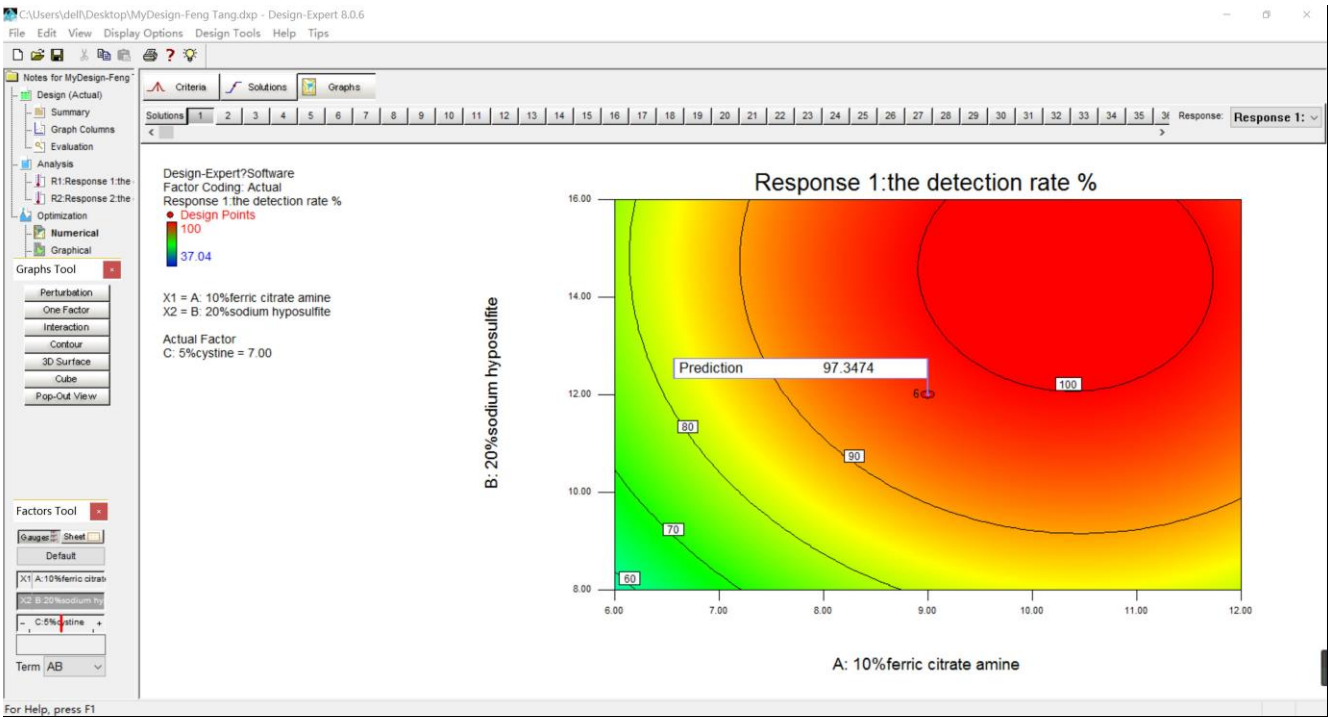
C:\Users\dell\Desktop\MyDesign-Feng Tang.dxp - Design-Expert 8.0.6
File Edit View Display Options Design Tools Help Tips
Criteria
Solutions
Graphs
Response:
Response 1:
Notes for MyDesign-Feng
Design (Actual)
Summary
Graph Columns
Evaluation
Analysis
R1:Response 1:the
R2:Response 2:the
Optimization
Numerical
Graphical
Graphs Tool
Perturbation
One Factor
Interaction
Contour
3D Surface
Cube
Pop-Out View
Factors Tool
Gauges
Sheet
Default
Term AB
Design-Expert?Software
Factor Coding: Actual
Response 1:the detection rate %
Design Points
100
37.04
X1 = A: 10%ferric citrate amine
X2 = B: 20%sodium hyposulfite
Actual Factor
C: 5%cystine = 7.00
Response 1:the detection rate %
Prediction 97.3474
B: 20%sodium hyposulfite
A: 10%ferric citrate amine
For Help, press F1

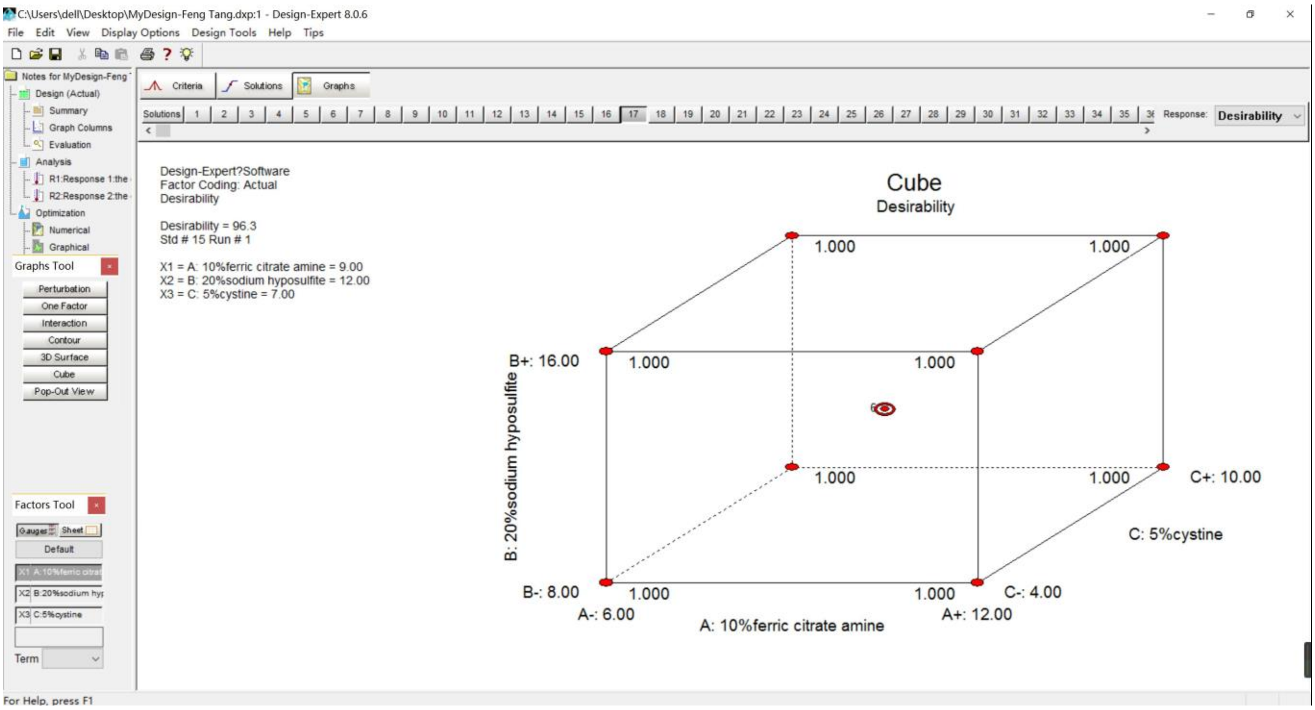
C:\Users\dell\Desktop\MyDesign-Feng Tang.dxp:1 - Design-Expert 8.0.6
File Edit View Display Options Design Tools Help Tips
Criteria
Solutions
Graphs
Response: Desirability
Design-Expert?Software
Factor Coding: Actual
Desirability
Desirability = 96.3
Std # 15 Run # 1
X1 = A: 10%ferric citrate amine = 9.00
X2 = B: 20%sodium hyposulfite = 12.00
X3 = C: 5%cystine = 7.00
Cube
Desirability
1.000
1.000
B+: 16.00
1.000
1.000
B: 20%sodium hyposulfite
1.000
1.000
C+: 10.00
C: 5%cystine
B-: 8.00
1.000
1.000
C-: 4.00
A-: 6.00
A: 10%ferric citrate amine
A+: 12.00
For Help, press F1

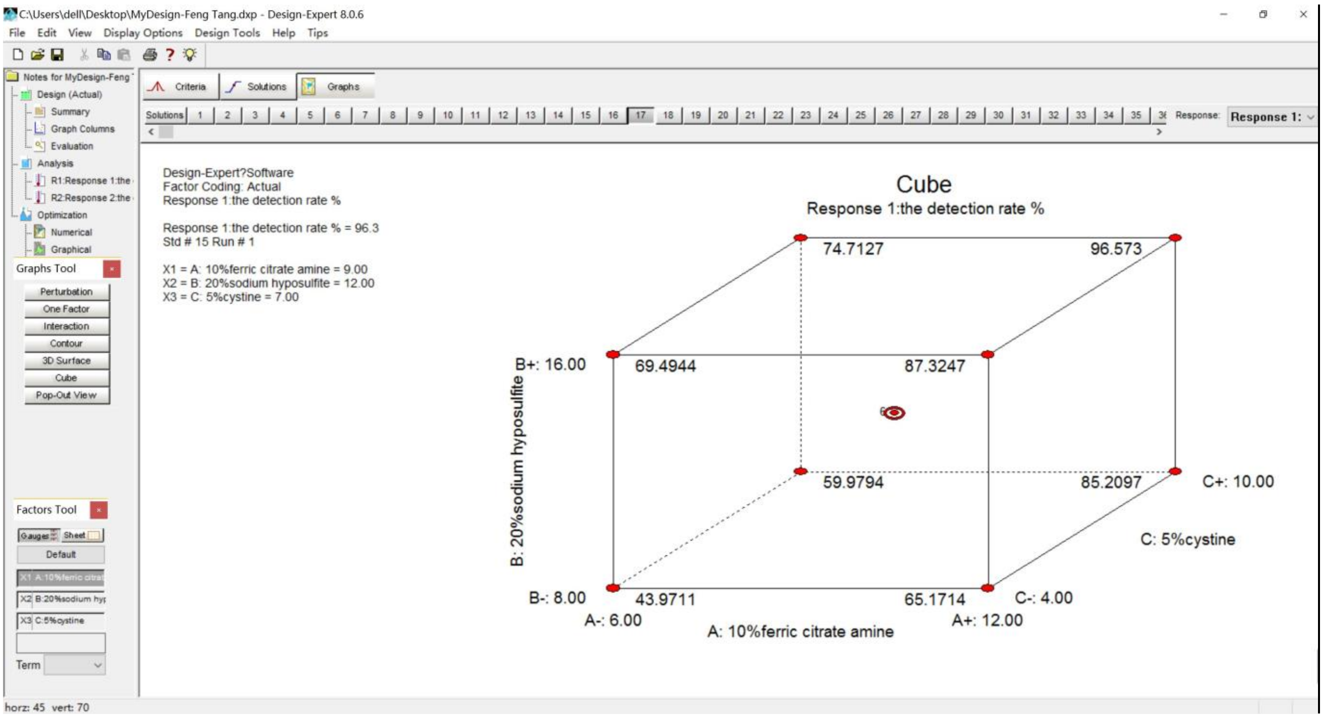
C:\Users\dell\Desktop\MyDesign-Feng Tang.dxp - Design-Expert 8.0.6
File Edit View Display Options Design Tools Help Tips
Criteria
Solutions
Graphs
Response: Response 1:
Design-Expert?Software
Factor Coding: Actual
Response 1:the detection rate %
Response 1:the detection rate % = 96.3
Std # 15 Run # 1
X1 = A: 10%ferric citrate amine = 9.00
X2 = B: 20%sodium hyposulfite = 12.00
X3 = C: 5%cystine = 7.00
Cube
Response 1:the detection rate %
74.7127
96.573
B+: 16.00
69.4944
87.3247
B: 20%sodium hyposulfite
59.9794
85.2097
C+: 10.00
C: 5%cystine
B-: 8.00
43.9711
65.1714
C-: 4.00
A-: 6.00
A: 10%ferric citrate amine
A+: 12.00
horz: 45 vert: 70

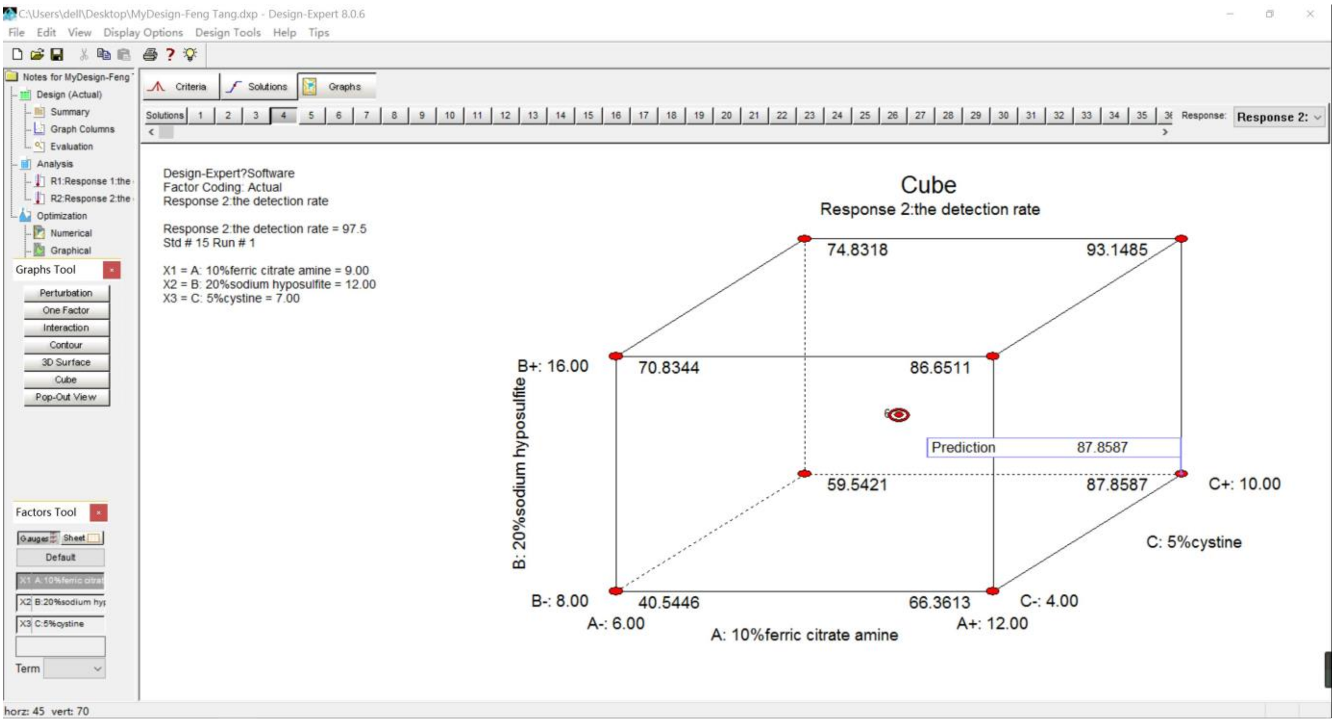
C:\Users\dell\Desktop\MyDesign-Feng Tang.dxp - Design-Expert 8.0.6
File Edit View Display Options Design Tools Help Tips
Criteria
Solutions
Graphs
Response: Response 2:
Design-Expert?Software
Factor Coding: Actual
Response 2:the detection rate
Response 2:the detection rate = 97.5
Std # 15 Run # 1
X1 = A: 10%ferric citrate amine = 9.00
X2 = B: 20%sodium hyposulfite = 12.00
X3 = C: 5%cystine = 7.00
Cube
Response 2:the detection rate
74.8318
93.1485
70.8344
86.6511
Prediction 87.8587
59.5421
87.8587
40.5446
66.3613
B+: 16.00
B-: 8.00
B: 20%sodium hyposulfite
A-: 6.00
A+: 12.00
A: 10%ferric citrate amine
C+: 10.00
C-: 4.00
C: 5%cystine
Graphs Tool
Perturbation
One Factor
Interaction
Contour
3D Surface
Cube
Pop-Out View
Factors Tool
Default
Term
horz: 45 vert: 70
