# Supplementary material for: Optimization of an efficient solid-phase enrichment medium for Salmonella detection using response surface methodology
Source: AMB Express. 2019 Jun 28;9:97. doi: 10.1186/s13568-019-0819-0 (PMC6598893; doi:10.1186/s13568-019-0819-0)
Supplement: Supplementary file 2 — Additional file 2. The predicted response for the nine standard Salmonella strains (Y1). [file 13568_2019_819_MOESM2_ESM.pdf]

Response 1 Response 1:the detection rate %

ANOVA for Response Surface Quadratic Model

Analysis of variance table [Partial sum of squares - Type III]

**Source**	**Sum of Squares**	**df**	**Mean Square**	**F Value**	**p-value Prob > F**	
Model	6080.75	9	675.64	13.76	0.0002	significant
A-10%ferric citrate amine	*1582.68*	*1*	*1582.68*	*32.23*	*0.0002*	
B-20%sodium hyposulfite	*1161.37*	*1*	*1161.37*	*23.65*	*0.0007*	
C-5%cystine	*544.48*	*1*	*544.48*	*11.09*	*0.0076*	
AB	*5.68*	*1*	*5.68*	*0.12*	*0.7408*	
AC	*8.12*	*1*	*8.12*	*0.17*	*0.6928*	
BC	*58.21*	*1*	*58.21*	*1.19*	*0.3018*	
A^2*1963.03*	*1*	*1963.03*	*39.98*	*< 0.0001*		
B^2*724.60*	*1*	*724.60*	*14.76*	*0.0033*		
C^2*481.61*	*1*	*481.61*	*9.81*	*0.0107*		
Residual	491.00	10	49.10			
Lack of Fit	*445.27*	*5*	*89.05*	*9.74*	*0.0130*	*significant*
Pure Error	*45.73*	*5*	*9.15*			
Cor Total	6571.75	19				

The Model F-value of 13.76 implies the model is significant. There is only a 0.02% chance that a "Model F-Value" this large could occur due to noise.

Values of "Prob > F" less than 0.0500 indicate model terms are significant.
In this case A, B, C, A^2, B^2, C^2 are significant model terms.
Values greater than 0.1000 indicate the model terms are not significant.
If there are many insignificant model terms (not counting those required to support hierarchy), model reduction may improve your model.

The "Lack of Fit F-value" of 9.74 implies the Lack of Fit is significant. There is only a 1.30% chance that a "Lack of Fit F-value" this large could occur due to noise.
Significant lack of fit is bad -- we want the model to fit.

Std. Dev.	7.01	R-Squared	0.9253
Mean	80.59	Adj R-Squared	0.8580
C.V. %	8.69	Pred R-Squared	0.4747
PRESS	3452.25	Adeq Precision	10.773

The "Pred R-Squared" of 0.4747 is not as close to the "Adj R-Squared" of 0.8580 as one might normally expect. This may indicate a large block effect or a possible problem with your model and/or data. Things to consider are model reduction, response transformation, outliers, etc.

"Adeq Precision" measures the signal to noise ratio. A ratio greater than 4 is desirable. Your ratio of 10.773 indicates an adequate signal. This model can be used to navigate the design space.

**Coefficient Factor**	**Estimate**	**Standard df**	**95% CI Error**	**95% CI Low**	**High**	**VIF**
Intercept	97.35	1	2.86	90.98	103.72	

A-10%ferric citrate amine10.77		1	1.90	6.54	14.99	1.00
B-20%sodium hyposulfite9.22		1	1.90	5.00	13.45	1.00
C-5%cystine	6.31	1	1.90	2.09	10.54	1.00
AB-0.84	1	2.48	-6.36	4.68	1.00	
AC1.01	1	2.48	-4.51	6.53	1.00	
BC-2.70	1	2.48	-8.22	2.82	1.00	
A^2-11.67	1	1.85	-15.78	-7.56	1.02	
B^2-7.09	1	1.85	-11.20	-2.98	1.02	
C^2-5.78	1	1.85	-9.89	-1.67	1.02	

Final Equation in Terms of Coded Factors:

Response 1:the detection rate % =

+97.35
+10.77 * A
+9.22 * B
+6.31 * C
-0.84 * A * B
+1.01 * A * C
-2.70 * B * C
-11.67 * A^2
-7.09 * B^2
-5.78 * C^2

Final Equation in Terms of Actual Factors:

Response 1:the detection rate % =

-197.09011
+26.98952 * 10%ferric citrate amine
+15.14709 * 20%sodium hyposulfite
+12.78726 * 5%cystine
-0.070208 * 10%ferric citrate amine * 20%sodium hyposulfite
+0.11194 * 10%ferric citrate amine * 5%cystine
-0.22479 * 20%sodium hyposulfite * 5%cystine
-1.29679 * 10%ferric citrate amine^2
-0.44318 * 20%sodium hyposulfite^2
-0.64232 * 5%cystine^2

The Diagnostics Case Statistics Report has been moved to the Diagnostics Node.
In the Diagnostics Node, Select Case Statistics from the View Menu.

Proceed to Diagnostic Plots (the next icon in progression). Be sure to look at the:

1) Normal probability plot of the studentized residuals to check for normality of residuals.
2) Studentized residuals versus predicted values to check for constant error.
3) Externally Studentized Residuals to look for outliers, i.e., influential values.
4) Box-Cox plot for power transformations.

If all the model statistics and diagnostic plots are OK, finish up with the Model Graphs icon.
